# Five-analyzer Johann spectrometer for hard X-ray photon-in/photon-out spectroscopy at the Inner Shell Spectroscopy beamline at NSLS-II: design, alignment and data acquisition

**DOI:** 10.1107/S1600577524009342

**Published:** 2024-10-30

**Authors:** Akhil Tayal, David Scott Coburn, Donald Abel, Max Rakitin, Oksana Ivashkevych, Jakub Wlodek, Dominik Wierzbicki, Weihe Xu, Evgeny Nazaretski, Eli Stavitski, Denis Leshchev

**Affiliations:** a National Synchrotron Light Source II Brookhaven National Laboratory Upton NY11973 USA; b AGH University of Science and Technology, Faculty of Energy and Fuels, Al. A. Mickiewicza 30, 30-059Cracow, Poland; ESRF – The European Synchrotron, France

**Keywords:** X-ray spectroscopy, X-ray emission spectrometers, Johann geometry

## Abstract

Here, a recently commissioned five-analyzer Johann spectrometer at the Inner Shell Spectroscopy beamline (8-ID) at the National Synchrotron Light Source II (NSLS-II) is presented. Designed for hard X-ray photon-in/photon-out spectroscopy, the spectrometer achieves a resolution in the 0.5–2 eV range, depending on the element and/or emission line, providing detailed insights into the local electronic and geometric structure of materials. It serves a diverse user community, including fields such as physical, chemical, biological, environmental and materials sciences. This article details the mechanical design, alignment procedures and data-acquisition scheme of the spectrometer, with a particular focus on the continuous asynchronous data-acquisition approach that significantly enhances experimental efficiency.

## Abstract 2

Here, the design, alignment and data acquisition of a five-analyzer Johann spectrometer at the Inner Shell Spectroscopy beamline at the National Synchrotron Light Source II are presented.

## 1. Introduction

Photon-in/photon-out techniques such as high-energy resolution fluorescence-detected (HERFD) X-ray absorption spectroscopy (XAS) (Hämäläinen *et al.*, 1991 ▸), X-ray emission spectroscopy (XES) (Gohshi *et al.*, 1982 ▸; Bergmann & Glatzel, 2009 ▸) and resonant inelastic X-ray scattering (RIXS) (de Groot, 2001 ▸) have become indispensable tools at synchrotron radiation facilities worldwide, and are being extensively used in diverse fields including materials science, chemistry, environmental science and life sciences (Bauer, 2014 ▸; de Groot, 2001 ▸; Guilherme Buzanich, 2022 ▸; Hämäläinen *et al.*, 1992 ▸; Szlachetko *et al.*, 2013 ▸). These techniques extend the capabilities of conventional XAS, which consists of X-ray absorption near-edge spectroscopy (XANES) and extended X-ray absorption fine structure (EXAFS), and is widely used to probe element-specific local structures (Bunker, 2010 ▸; Iwasawa *et al.*, 2017 ▸). Detection of X-ray fluorescence lines with higher resolution in HERFD-XAS allows one to obtain well resolved near-edge features in the XANES region, which are otherwise obscured due to core-hole broadening (Hämäläinen *et al.*, 1991 ▸). Furthermore, one can resolve overlapping edges in multi-element alloy systems, allowing for range-extended EXAFS, which has recently become popular for many catalysis applications (Asakura *et al.*, 2018 ▸; Yano *et al.*, 2005 ▸) and materials science (Lafuerza *et al.*, 2016 ▸, 2020 ▸). XES provides complementary information on the elements’ charge, spin state (Hämäläinen *et al.*, 1992 ▸; Vankó *et al.*, 2006 ▸) and ligand type (Reinhard *et al.*, 2023 ▸; Pollock & DeBeer, 2015 ▸). Mapping out the X-ray emission intensity as a function of both emission and incident photon energies around the pre-edge resonances, as in RIXS, helps one to probe the covalency of the metal–ligand bonds (Baker *et al.*, 2017 ▸), disentangle different ligand-field and charge transfer transitions (Lundberg & Wernet, 2020 ▸), investigate electron localization dynamics (Amidani *et al.*, 2015 ▸), and obtain complementary insights into the oxidation state (Castillo *et al.*, 2021 ▸). Due to the advantages in gaining information on electronic properties, local coordination, geometry and disorder, irrespective of material form, these techniques are widely utilized to investigate complex materials relevant to catalysis (Albrahim *et al.*, 2021 ▸; Porter *et al.*, 2023 ▸; Boubnov *et al.*, 2014 ▸), batteries (Kankanallu *et al.*, 2023 ▸; Nishimura *et al.*, 2019 ▸), energy materials (Ebrahim *et al.*, 2020 ▸; Yang *et al.*, 2023 ▸) and environmental applications (Pincus *et al.*, 2023 ▸; Manceau *et al.*, 2021 ▸, 2015 ▸). In addition, the increasing flux at new and upgraded synchrotron facilities has contributed immensely to realizing complex *operando* experiments with these techniques (Feijóo *et al.*, 2023 ▸).

In photon-in/photon-out spectroscopy, fluorescence X-rays are detected using bent crystal analyzers (CAs), which disperse and/or focus emitted photons in a narrow energy range (DuMond, 1947 ▸). Curved CAs show higher efficiency compared with flat CAs due to larger solid-angle coverage. Several spectrometers utilizing bent CAs were developed at different synchrotron facilities. The spectrometer generally consists of the X-ray source, usually a sample, a bent CA and a detector, which are placed either in Johann or von Hamos geometry. In the von Hamos geometry, the source is placed on the longitudinal axis of a cylindrically bent CA, which refocuses and disperses the X-rays on a 2D position-sensitive detector. This arrangement allows one to image an emission line without moving any of the spectrometer parts and is suitable for fast time-resolved experiments (Alonso-Mori *et al.*, 2012 ▸; Abraham *et al.*, 2019 ▸). On the other hand, von Hamos spectrometers tend to offer a relatively low signal-to-background ratio due to the limited CA surface area that disperses the photons, thus limiting the application of a spectrometer to dilute systems. In the Johann geometry, one uses a spherically bent CA (SBCA), in which the entire surface diffracts photons in a narrow energy range, thus offering larger solid-angle coverage for a given energy and better suited for measuring dilute samples. At the same time, the Johann-type spectrometer requires point-to-point scanning and more complex mechanics. Regardless of the geometry, an additional increase in the solid angle is achieved by employing multiple SBCAs, thus enabling experimentation on more dilute systems, albeit at the cost of more complicated mechanical motion solutions, particularly for the Johann geometry. The ability of the photon-in/photon-out techniques to provide deep insights into material properties has inspired the construction of many instruments at different synchrotron facilities (Brookes *et al.*, 2018 ▸; Duan *et al.*, 2017 ▸; Edwards *et al.*, 2022 ▸; Evgueni *et al.*, 2009 ▸; Glatzel *et al.*, 2021 ▸; Gog *et al.*, 2009 ▸; Hayama *et al.*, 2021 ▸; Honkanen *et al.*, 2019 ▸; Kalinko *et al.*, 2020 ▸; Kleymenov *et al.*, 2011 ▸; Kvashnina & Scheinost, 2016 ▸, Llorens *et al.*, 2012 ▸; Mattern *et al.*, 2012 ▸; Sokaras *et al.*, 2012 ▸; Szlachetko *et al.*, 2013 ▸; Uwe & Stephen, 1998 ▸), as well as benchtop instrumentation (Jahrman *et al.*, 2019 ▸; Zimmermann *et al.*, 2020 ▸; Seidler *et al.*, 2014 ▸). Continuously increasing demand for such instrumentation due to growth of the user base, as well as progress in the development of user-friendly software for data analysis (Newville, 2013 ▸) and theoretical modeling (Rehr *et al.*, 2010 ▸; Bunău *et al.*, 2024 ▸; Neese, 2012 ▸), calls for further development of high-resolution spectrometers to meet the needs of the research community.

In this article, we present a five-crystal Johann-type spectrometer developed at the Inner Shell Spectroscopy (ISS) beamline of the National Synchrotron Light Source II (NSLS-II). The article provides a detailed description of the spectrometer’s mechanics and motion control, the instrument alignment procedures, as well as recent developments in data acquisition with a particular emphasis on the continuous scanning of both incident and emitted photon energies.

## 2. Spectrometer description

The ISS beamline features a damping wiggler as the photon source and a servo motor-driven rapid-scanning cryogenically cooled Si(111) monochromator. The beamline’s energy coverage range is between 4.9 and 33 keV and its flux is up to 5 × 10^13^ photons s^−1^ at 10 keV. The beam is focused to a 1 mm spot at the sample position. Further focusing of the beam, required for high-resolution experiments, is accomplished by a polycapillary optic upstream of the sample, which focuses the X-rays to a round Gaussian spot with a full width at half-maximum (FWHM) of 50–200 µm, depending on the specific optic and the distance to the sample. Further details of the beamline design can be found in the literature (Leshchev *et al.*, 2022 ▸).

**Figure 1:**
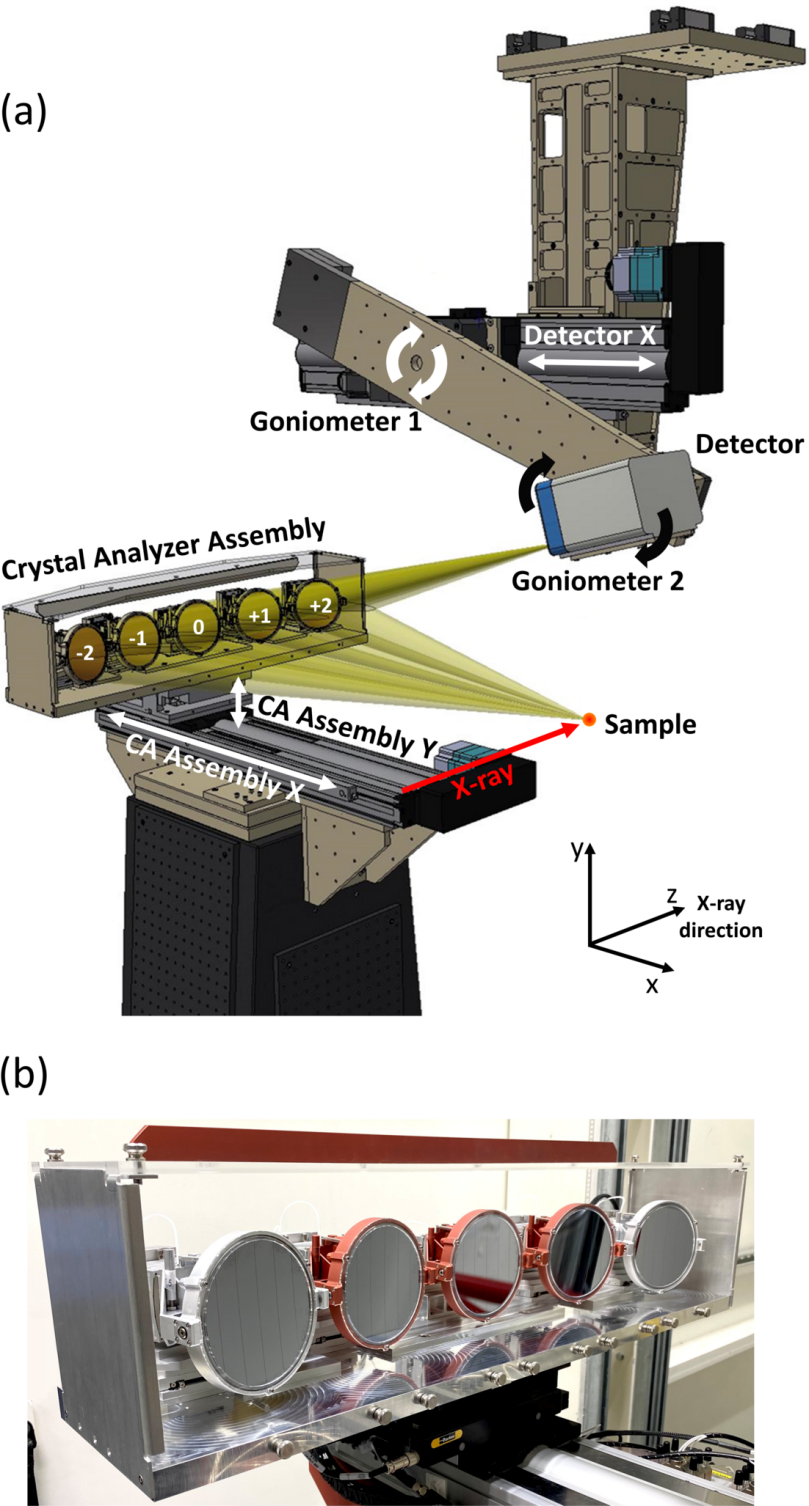
The ISS spectrometer design. (*a*) A design drawing showing the CA assembly, its *X*–*Y* motion, the detector arm with two goniometers and the arm’s *X* motion. The five CAs are labeled as shown with respect to the X-ray beam direction. The beamline coordinate system is shown for reference. (*b*) A photograph of the analyzer assembly taken at the beamline.

The ISS spectrometer mechanical design and a photograph of the SBCA assembly are shown in Fig. 1 ▸. The spectrometer operates with up to five SBCAs that are placed on intersecting Rowland circles. The SBCAs are labeled with indexes *i* = (0, ±1, ±2) as shown in Fig. 1 ▸. The SBCA assembly is positioned such that the central SBCA (*i* = 0) is located in a horizontal plane and perpendicular to the incident beam to minimize the elastic background. The SBCA assembly is placed on top of the motor stack with translation stages for *x* and *y* motions. To minimize vibrations from the environment, the SBCA assembly with the motion stack is installed on a solid granite pedestal grouted to the floor. The SBCA assembly consists of a common plate holding individual SBCA mechanical stacks, which are described below. The detector motion is implemented using two rotation stages and a linear stage (see Section S1 of the supporting information). To scan the emission energy, the spectrometer parts are moved according to the standard equations describing the Johann geometry (Glatzel *et al.*, 2021 ▸; Kvashnina & Scheinost, 2016 ▸). The spectrometer mechanics are designed to operate with Rowland circle radii between 500 and 1000 mm, and cover Bragg angles between 65 and 90°. In practice, the higher limit on the Bragg angle is determined by the sample environment and whether it collides with the detector. To minimize absorption and diffuse scattering of the emitted X-rays in air, a helium enclosure, tailored to specific Rowland circle radii and Bragg angle ranges, are placed between the sample, the SBCA assembly and the detector.

**Figure 2:**
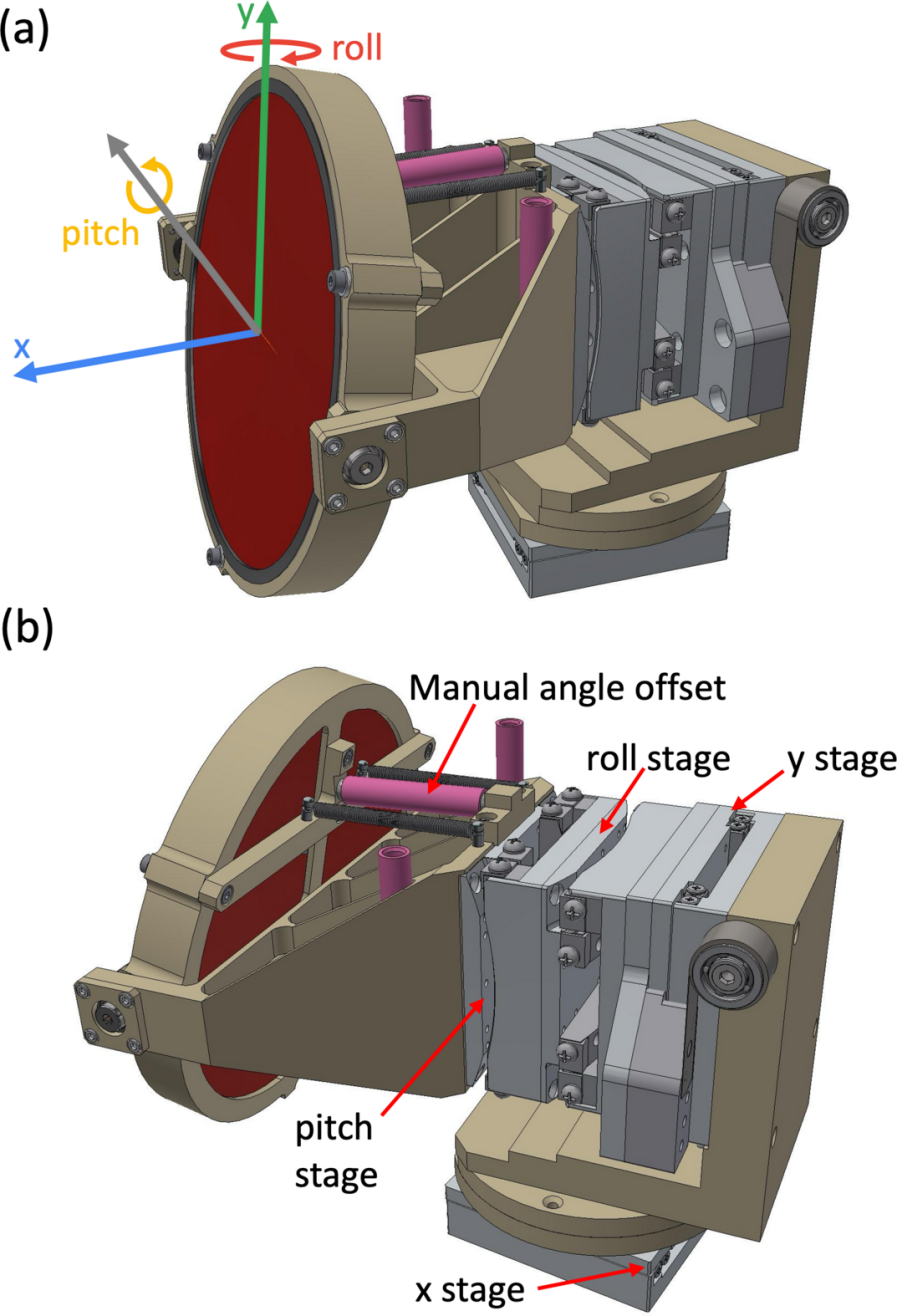
The design of the SBCA mechanical stack. (*a*) The mechanical stack with four motion axes *viz*. *x*, *y*, pitch and roll shown. (*b*) The location of the four motion stages and a spring-loaded mechanism with a hollow stud (magenta) used to adjust the angle offset manually. Hollow studs with different lengths (magenta) are available for different angular offsets and are kept on the sides of the stacks.

The mechanical stack for SBCAs with *i* = (±1, ±2) is shown in Fig. 2 ▸ and consists of two tilt stages (SmarAct CGO series) to scan SBCA pitch and roll, and two translation stages (SmarAct CLS series) for vertical and horizontal motion. The central SBCA (*i* = 0) mechanical stack omits linear stages. The tilt stages have a nominal angular travel range of ±5° and the linear stages have a nominal travel range of ±15 mm. The choice of the stages relied on our previous instrumentation development experiences (Nazaretski *et al.*, 2022 ▸). The compact form factor of the stacks opens an opportunity to add another row of SBCAs on top of the current one in the future, which will increase the solid angle captured by the instrument. In addition, the stages are suitable for rapid scanning, with speeds of up to 4° s^−1^ and 20 mm s^−1^ for tilt and translation stages, respectively, which can further increase data-acquisition efficiency. To cover the full range of Bragg angles, each SBCA stack includes a spring-loaded mechanism that allows for a manual offset of the crystal pitch [Fig. 2 ▸(*b*)]. The position and orientation of the individual stacks with *i* = (±1, ±2) can be manually adjusted to predefined positions for operations at Rowland circle radii between 500 and 1000 mm. When operating at Bragg angles below 70° at 500 mm Rowland circle radius, additional vertical offsets are installed for the side SBCAs with *i* = ±2 to enable their correct tracking of the Johann geometry. The SBCA mounts accommodate SBCAs supported on round substrates with a diameter of 10 cm. At ISS, we use SBCAs from several vendors, such as XRS Tech, Luxium Solutions and AlpyX. The currently available analyzers and their corresponding energy ranges are listed in Section S2.

To avoid loss of resolution due to misalignment between the spectrometer components, reproducibility of the mechanical motions must be ensured; in particular, for the SBCA positioning. With piezoelectric linear stages exhibiting positioning precision on the sub-micrometre level, the overall translational repeatability is determined primarily by the large linear stages for SBCA assembly and the detector, which have a repeatability of 5 µm. The angular-motion repeatability for SBCA pitch stages was measured to be within ±0.2 mdeg using the optical interferometer setup described in Section S3. Overall, accounting for thermal drifts, the angular positioning of SBCAs is estimated to be consistent within 0.5 mdeg precision.

The spectrometer detector mount can support two kinds of detectors: a Pilatus 100K 2D pixelated area detector (Dectris), and a silicon drift detector (SDD) system consisting of a Vortex-60EX detector with 80 mm^2^ active area (Hitachi High-Tech Science America, Inc.) and an Xspress 3X readout electronics module (Quantum Detectors Ltd). The choice of the detector and, as in the case of Pilatus 100K, the specific mounting orientation depends on the experiment and the constraints provided by the sample environment. In general, SDD is the detector of choice in the case of ultra-dilute samples and/or samples exhibiting a strong background. For less challenging systems, Pilatus 100K offers sufficient performance and, in our experience, offers beamline users an additional advantage of more intuitive operations and troubleshooting of the spectrometer due to the detector’s ability to visualize the individual SBCA reflections. The latter also streamlines the spectrometer alignment, as shown below. Moreover, we observed that the Bragg spot on the detector covers an area of ∼75 mm^2^, spanning about 50 × 50 pixels, with potential for smaller coverage. This coverage is comparable to the available size of an SDD and offers a similar spatial resolution. However, the Pilatus detector has the advantage of being gated down to 250 ns, which can be leveraged for pump–probe experiments in the future.

## 3. Spectrometer alignment

The spectrometer alignment consists of three main steps: pre-alignment using an optical laser, scanning of the SBCA resolution as a function of Rowland circle radius *R* and calibration of individual SBCA Bragg angles to ensure that all SBCAs diffract X-rays at the same energy. During installation, the nominal positioning of the spectrometer parts is verified using a laser distance meter, which is then used as a starting point for the following alignment procedures.

At the beginning of the spectrometer operations, the appropriate SBCAs are installed into the crystal mounts, and the manual angular offsets and SBCA assembly mechanical stages are set to their nominal positions corresponding to the selected SBCA curvature and the Bragg angle of 90°. To adjust the spectrometer to the X-ray beam height, a level laser is set at the height of the beam. The center of SBCA *i* = 0 then aligns to the laser level by adjusting the crystal assembly height. The remaining crystals are aligned in a similar fashion. This alignment method allows one to set the height within ∼0.3 mm; any errors from this procedure are corrected during the spectrometer calibration. Then, a source of visible light is placed into the X-ray beam position by focusing a laser pointer onto a well scattering surface, such as a piece of paper, placed in the sample holder. The angular motions on each SBCA stack are then adjusted such that the reflected light from the polished SBCA surface is reflected back into the source spot, which is judged by a visual inspection using sample cameras. The positions of SBCA stages are then saved as a reference 90° backscattering configuration. Then, all SBCAs, the detector arm, as well as manual angle offsets for each SBCA, are set to the calculated positions for the emission line of interest. The motor positions are calculated using the equations describing the Johann geometry that can be found in the literature (Glatzel *et al.*, 2021 ▸; Kvashnina & Scheinost, 2016 ▸). Additional visual inspection is performed at this step to ensure that each SBCA re-images the source onto the detector sensor, and SBCA pitch and roll angles are adjusted, if necessary. We find that this step is mostly necessary only during changes between the spectrometer configurations with different *R* values, whereas swapping SBCAs in the configuration with the same *R* does not drastically affect the mechanical positioning, in which case this step is optional.

In the second step of alignment, spectrometer resolution is optimized with the X-ray beam. Although the nominal SBCA radius of curvature is set during manufacturing by the substrate, the actual curvature often deviates from the nominal value due to imperfections in the substrate shape and the stress induced by the bonding process, which in turn affects the SBCA resolution. Thus, the true radius of curvature needs to be determined for each SBCA to obtain the best possible resolution. During this alignment step, the true curvature of the SBCAs is determined by varying the Rowland circle radius of the spectrometer within ±10–15 mm of the nominal value; at each Rowland circle radius, the instrument response function of the spectrometer is determined using either elastic scattering or emission signal, as discussed below.

In this alignment step, we use the Pilatus 100K detector, which allows for convenient visualization of individual reflections from each SBCA. Additionally, scans that nominally require coordinated motion of SBCA and detector can be performed faster, since the detector has a relatively large sensitive area and thus can be held in a constant position. During this step, in order to establish a region of interest (ROI) on the Pilatus detector, we place an appropriate reference foil into the sample position and illuminate it with X-rays with energy set above the absorption edge of interest. Upon exposure, Pilatus images show reflections produced by each SBCA. Minor SBCA pitch adjustments are made to tune SBCAs to the maximum of the relevant emission line so that the SBCA Bragg angles coarsely agree with each other. Additional SBCA roll adjustments are made to ensure that SBCA reflections are positioned within a relatively small ROI on the Pilatus sensitive area, thus preparing the setup for the scanning.

The SBCA curvature can be determined in two different ways: namely, by characterizing the broadening of the elastic peak or the emission line as a function of Rowland circle radius. In the former case, the elastic signal intensity is scanned as a function of the incident beam energy while the SBCA Bragg angle is kept constant; in the second case, the emission signal intensity is scanned as a function of the SBCA Bragg angle while the incident beam energy is kept constant above the absorption edge of the foil. A recent study on an X-ray emission spectrometer developed at the European Synchrotron Radiation Facility demonstrated the utility of emission scans for alignment in the tender X-ray regime (Rovezzi *et al.*, 2020 ▸). At ISS, we have incorporated both methods into the alignment routines; the specific choice of method depends on the experiment at hand. For instance, in the case of low-*Z* elements, such as 3*d* metals, the *K*α and *K*β emission lines tend to be relatively narrow and thus can be used for the characterization of SBCA curvature. The additional benefit of the high intensity of these lines also allows for a more rapid alignment. Conversely, high-*Z* elements, such as 5*d* metals, tend to have broad emission lines, and thus scanning the elastic peak is better suited for determining the SBCA curvature. Regardless of the approach, a metric derived from a scan, such as peak maximum intensity or FWHM, needs to be computed to determine the true SBCA curvature. At ISS, we generally consider both these metrics; however, we find that peak maximum intensity is more consistent when comparing elastic and emission approaches, as discussed below. This might be due to the FWHM metric becoming less representative of the peak broadening in cases where the instrument response function becomes asymmetric and/or contains low-intensity tails due to, for example, operating the spectrometer at low Bragg angles. These effects have been documented before and alternative figures of merit, combining both FWHM and peak maximum intensity, were proposed (Glatzel *et al.*, 2021 ▸; Rovezzi *et al.*, 2020 ▸). In practice, analyzer edge masking can be used to sharpen the spectrometer response function and reduce ambiguities associated with the spectrometer alignment.

**Figure 3:**
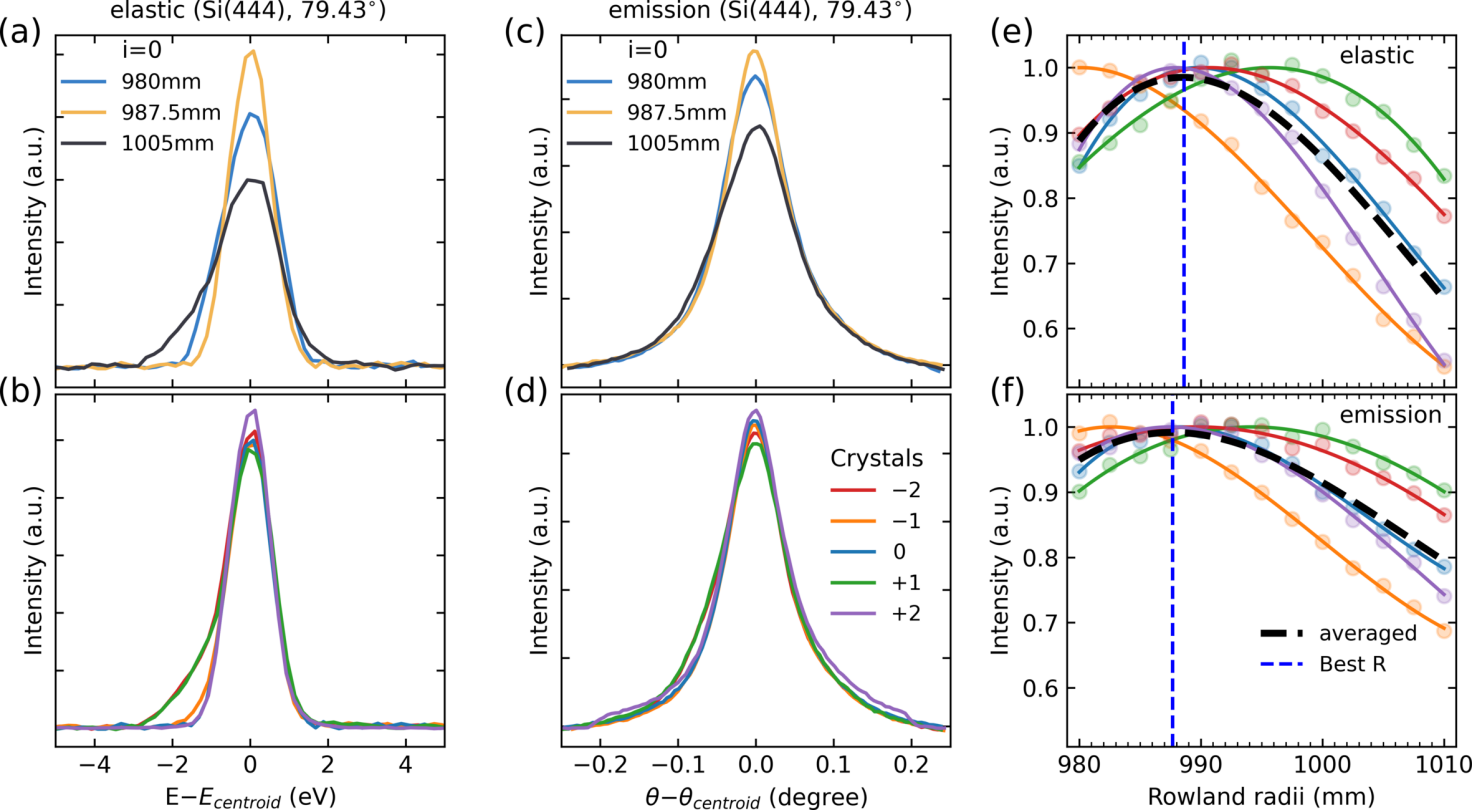
(*a*, *b*) Elastic and (*c*, *d*) emission scans for the alignment of the five-analyzer spectrometer with a nominal radius of curvature of 1000 mm. The elastic scans were performed on water and emission scans were performed on Cu foil, using the fourth reflection of Si(111) crystal with the 79.43° Bragg angle. (*a*) Elastic peaks measured at selected values of the Rowland circle radius, *R*, showing that large deviations from the optimal value result in peak broadening and reduction in the peak height. (*b*) Elastic peaks measured at the optimal *R* for all analyzers. The peaks for each SBCA are normalized by the peak maximum intensity observed in the corresponding scan series (*c*, *d*). In (*c*, *d*) a similar alignment procedure to the one used for the elastic scans is followed, but these scans display the Cu *K*α_1_ emission peak. (*e*) The elastic peak maximum intensity as a function of *R* for all SBCAs. Colored circles show the values obtained from each scan. Solid lines show the third-order polynomial fit of the data. The average of the polynomial fits of all SBCA data (dashed black line) is used to determine the position of the curve maximum, which is used as the optimal *R* value (dashed blue line). For clarity, all the curves are normalized by the maximum of the corresponding polynomial fits. Panel (*f*) is the same as (*e*), but for the Cu *K*α_1_ emission peak. Panels (*b*), (*d*), (*e*), and (*f*) use the same color scheme to show SBCA-specific data.

Fig. 3 ▸ shows the results of the SBCA resolution scanning as a function of Rowland circle radius, performed both using elastic peak and emission line scanning for operations at 8046 eV, *i.e.* in the vicinity of the Cu *K*α_1_ emission line peak, with five Si(444) crystals with nominal 1000 mm curvature radius at a Bragg angle of 79.43°. The elastic scans were performed at 8046 eV using a 1 mm Kapton tube with de­ionized water as a sample. To scan the emission line, Cu foil was placed in the sample position, while the incident X-ray energy was kept at 9000 eV. The emission line scans were performed by scanning the crystal pitch, with all other SBCA degrees of freedom kept constant. This approach approximates the true motion along the Rowland circle needed to change the SBCA Bragg angle; however, given the small angular window required to estimate the emission peak width, ∼0.1° in this case, the systematic error due to approximation is negligible. In addition, the SBCA’s simplified motion allows one to execute scans in a continuous fashion, which improves the scanning speed and shortens the total alignment time. Both elastic and emission signal intensities were calculated by integrating counts in appropriate ROIs of the detector and normalizing the signal by the incident beam intensity measured with an ion chamber. The results show that varying the Rowland circle radius to extremely low or high values leads to a drastic decrease in the peak maximum intensity and broadening for both elastic and emission scans [Figs. 3 ▸(*a*) and 3 ▸(*c*)]. Based on the peak maximum intensity of elastic and emission signals as a function of *R*, we observe that each SBCA resolution exhibits a distinct maximum that reflects the true curvature of each SBCA. Inspecting the data for individual SBCAs shows that the Rowland circle radius corresponding to the optimal resolution agrees between elastic and emission scans indicating equivalence of the approaches. A comparison of the optimal *R* for individual SBCAs determined based on the FWHM metric also shows overall fair agreement between the approaches, albeit with some differences for specific SBCAs (see Section S4). To compute the Rowland circle radius corresponding to the global optimum, we fit individual SBCA maximum peak intensity dependencies on *R* to a third-order polynomial, sum them up and determine the global maximum [black and blue dashed lines in Figs. 3 ▸(*e*) and 3 ▸(*f*)]. Once the optimal Rowland circle radius is determined, SBCAs are positioned accordingly and the Rowland circle radius value is kept constant during scanning of the emission energy. Inspection of the elastic and emission peaks measured at the global optimal *R* shows a fair agreement between different SBCAs, although some residual differences between responses may arise due to variations in the SBCA quality, as well as individual SBCA curvatures differing from the global optimal *R* [Figs. 3 ▸(*b*) and 3 ▸(*d*)].

In the third step of the alignment, we must ensure that all SBCAs are positioned at the same Bragg angle. This is usually done by setting the incident X-ray energy to the nominal spectrometer energy, followed by scanning the elastic signal as a function of the Bragg angle for each SBCA individually. Based on these scans, the effective Bragg angle corresponding to the set energy is selected as the peak maximum. As discussed above, at lower Bragg angles the elastic peak tends to be asymmetric, and thus the peak maximum position becomes less representative of the effective energy. Therefore, other measures, such as the peak center of mass or the mean value of the half-maxima energies (Glatzel *et al.*, 2021 ▸), should be used. In addition to the elastic scans, emission line peaks can also be used for Bragg angle calibration, provided that the line peaks are sufficiently sharp, such as in the case of 3*d* metals.

**Figure 4:**
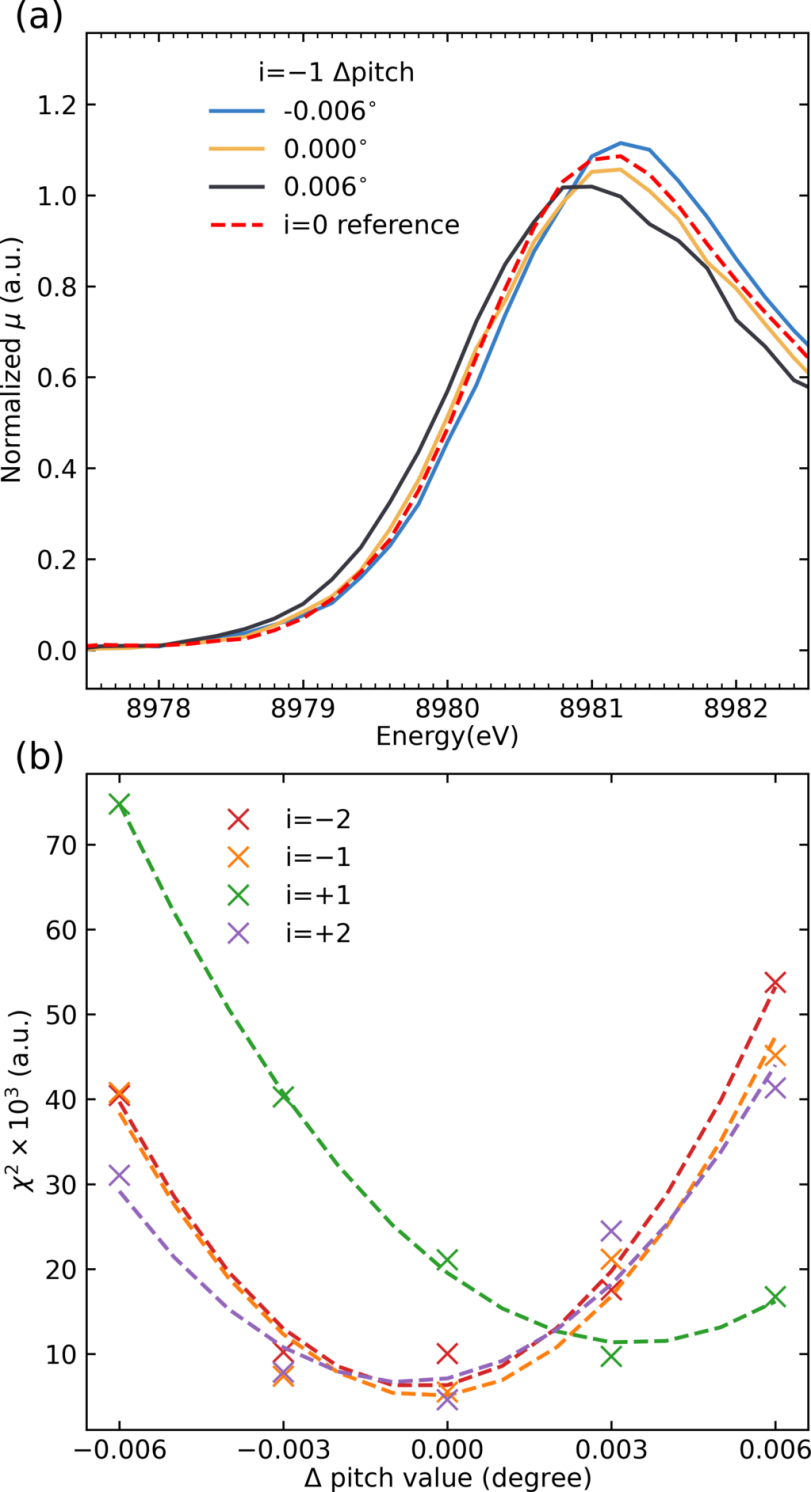
SBCA Bragg angle calibration with HERFD-XANES scans performed at different SBCA pitch values. (*a*) Selected alignment scans, performed on a dilute CuO sample, for SBCAs with *i* = +1 at different pitch values are compared with the HERFD-XANES measured for the *i* = 0 crystal. The pitch values are indicated as deviations from the values corresponding to the maximum of the elastic peak. (*b*) The sum of squared residuals, χ^2^ value, between HERFD-XANES data recorded for SBCAs with *i* = ±1, ±2 and the SBCA with *i* = 0 as a function of SBCA pitch-angle deviation. The dashed lines are second-order polynomial fits to the data.

As an additional way to verify that the individual SBCAs are calibrated to the same Bragg angle, we implemented a procedure based on scanning HERFD-XANES signals from a sample. The procedure is based on the fact that the edge position in a HERFD-XANES scan depends on the specific emission energy measured by a spectrometer (Lafuerza *et al.*, 2020 ▸; Glatzel *et al.*, 2013 ▸). Here, each SBCA pitch is initially positioned according to the maximum of the elastic peak. A HERFD-XANES scan is then performed using one of the crystals, typically the central SBCA (*i* = 0), which is then used as a reference for calibration of the remaining crystals. A set of HERFD-XANES scans is then performed for each SBCA (*i* = ±1, ±2) at different SBCA pitch positions within ±5–10 mdeg of the original setting. The sets of HERFD-XANES spectra are compared with the reference spectrum for *i* = 0 and the pitch angle values producing the best agreement in terms of least squares are chosen for each set. Following this procedure, all SBCAs must produce consistent HERFD-XANES scans indicating good agreement between individual SBCA Bragg angles. Fig. 4 ▸ shows the HERFD-XANES scans taken on a sample pellet with 1 wt% copper oxide (CuO) diluted in boron nitride. The data were recorded following a preliminary alignment of SBCAs based on the elastic peak measured from water. The individual HERFD-XANES spectra for SBCAs with *i* = ±1, ±2 show a good agreement with the spectrum recorded for the central SBCA, without any additional adjustments to the crystal pitch. However, for the SBCA with *i* = −1 it was found that the crystal pitch should be adjusted by 3 mdeg, which corresponds to ∼0.08 eV for this analyzer configuration. Given that the adjustment is smaller than the elastic peak FWHM of 1.5 eV, the result demonstrates the high sensitivity of the method to misalignment between the crystals. This approach is particularly beneficial when working at lower Bragg angles where assigning peak position becomes more ambiguous due to elastic peak asymmetry, as mentioned above. This procedure should be performed on dilute samples; in concentrated samples, due to variation in the emission exit angles from the sample, HERFD-XANES spectra measured by individual SBCAs can undergo different distortions due to self-absorption, potentially biasing the results.

Following the alignment, all motor position offsets are stored for this spectrometer energy and used as a reference point for the spectrometer motion control. We find that, due to imperfections of the mechanics, for reliable scanning of the emission energy, it is necessary to calibrate the SBCA Bragg angle at two energies. Thus, following calibration of the spectrometer at the first energy, the spectrometer is then moved to another energy, and the third step of the alignment is repeated. After recording two sets of motor position offsets for two energies, the offsets are interpolated as a function of Bragg angle. Naturally, this additional calibration is needed only for experiments that need scanning of the emission energy, *i.e.* XES and RIXS. The choice of reference energies at which the spectrometer should be calibrated is, in principle, arbitrary. With that, the best practice is to choose the energies representative of the emission energy range performed in an experiment. For instance, for experiments on 3*d* metals focusing on *K*α emission, we calibrate the spectrometer at energies corresponding to the *K*α_1_ and *K*α_2_ peaks. Likewise, for experiments on 3*d* metals focusing on *K*β and valence-to-core lines, we calibrate at the mainline *K*β_1,3_ and valence-to-core *K*β_2,5_ peak energies. After calibrating SBCAs to the same Bragg angle, we perform elastic scans for each SBCA individually to characterize the overall resolution of the spectrometer. The spectrometer resolution is estimated as an average of elastic peak FWHMs measured using each SBCA individually. The future commissioning of a high-resolution monochromator at ISS will help to better estimate the true energy resolution of the spectrometer.

Depending on the needs of the experiment, an appropriate detector, either Pilatus or SDD, is installed. When swapping from Pilatus to SDD, one needs to run a few additional scans to adjust the detector positioning and update the appropriate motor position offsets. When using Pilatus, additional shielding (250 µm tin foil) must be installed to minimize the background scattering reaching the detector. The alignment process described above is intended to be carried out once prior to the experiment, and the process takes ∼30–45 min depending on the specific requirements of the experiment and the initial state of the spectrometer. The evaluation of alignment data is performed in real time during measurements and is integrated within the alignment scan routine. Pre-alignment using the laser does not need to be repeated before each experiment, provided that the spectrometer configuration remains largely unchanged.

### 3.1. Effects of sample crystallinity and focusing optics on elastic line scans

In practice, we discovered that aligning the spectrometer using elastic scattering can often be unreliable. Variations in peak position and FWHM, influenced by sample crystallinity, can introduce biases when estimating the spectrometer resolution based on the analyzer response function. These inconsistencies can affect the accuracy of measurements, highlighting the need for careful consideration for alignment using elastic lines.

To illustrate this variation, we measured the elastic scattering peak signal from a set of crystalline samples, including Cu foil, CeO_2_, LaB_6_ and zeolite ZSM-5, and compared it with liquid water, which produces only diffuse scattering. The data and analysis are summarized in Section S5. For the same SBCA, the position of the peak maximum and FWHM was found to vary up to 0.1 eV and 0.2 eV, respectively. While relatively small, these variations can bias the analysis of XES data, especially when results hinge on small spectral differences between samples. To gain a better insight into the origin of these variations, we measured the scattering patterns produced by each sample on the surface of SBCAs with *i* = 0, +1, +2 by placing a Pilatus detector in front of each SBCA, as described in Section S6. The data show that the illumination of the SBCA surface depends on the specifics of the scattering pattern produced by each sample. Given that the effective Bragg angle varies across the SBCA surface due to bending, strain and miscut, the resulting elastic peak intensity distribution will depend on the way the crystal is illuminated and, therefore, the sample. These diffraction data were collected with the beam focused by a polycapillary optic, which produces a highly divergent beam. When focusing at the sample position is achieved using other optics, such as Kirkpatrick–Baez mirror pairs used at many other beamlines, the beam gets more collimated and thus the non-uniformity of the SBCA illumination is further exacerbated. Indeed, the scattering data collected without using the polycapillary optic show that the diffraction peaks become more defined (Section S6).

The observed dependence of the SBCA response function on the sample diffraction has several implications for the spectrometer operations. First, the elastic peak position can be a poor indicator of consistent spectrometer calibration when comparing crystalline samples with different structures. Thus, the stability and consistency of the spectrometer calibration should be verified using non-crystalline samples or samples that produce similar diffraction patterns. Second, the non-uniform illumination of the SBCA due to diffraction may affect the Rowland circle radius value corresponding to the SBCA’s optimal resolution. To see the extent of this effect, we performed a series of elastic scans on water and Cu foil while varying the Rowland circle radius (Section S4). We observe that both datasets show similar optimal points, even for SBCAs that are illuminated non-uniformly due to diffraction. While this indicates that SBCA bending is homogeneous across its surface, at least for this set of analyzers, we cannot rule out the possibility of this bias in the case of different SBCAs or different crystalline samples. Third, in the case of spectrometers with multiple SBCAs, calibration of the individual SBCA Bragg angles, as is described above in the third step of the alignment procedure, can become biased when done using crystalline samples due to the differences in the diffraction patterns cast on each SBCA surface. In this situation, the HERFD-XANES procedure for verifying individual SBCA calibration is necessary. Finally, characterization of the spectrometer resolution based on elastic scattering peak should be done using non-crystalline samples, since diffusely scattered X-rays yield a more uniform illumination of the SBCA surface. Overall, we find that, when operating the spectrometer, it is preferred to use emission signals to optimize or characterize its performance. When emission signals are weak or arise from broad lines, elastic signals from diffusely scattering samples should be used.

## 4. Spectrometer controls

The spectrometer controls are implemented using several software packages. The basic communications between the motors and the detectors are implemented using the *Experimental Physics and Industrial Control System* (*EPICS*) interface. The data acquisition for Pilatus 100K and Xspress3 detectors is configured using the *areaDetector EPICS* plugin (Rivers *et al.*, 2010 ▸). Scanning and general spectrometer motion controls are implemented using the *Ophyd* (https://blueskyproject.io/ophyd) and *Bluesky* (https://blueskyproject.io/bluesky) libraries (Allan *et al.*, 2019 ▸; Arkilic *et al.*, 2017 ▸). The high-level controls of the setup are implemented in the beamline Python/Qt-based graphical user interface (GUI). The GUI contains widgets for the configuration of the alignment scan sequences and alignment data visualization. The user-facing widgets allow convenient operation of the spectrometer using the emission energy pseudo-motor, which is translated into individual motor movements.

The implemented control system also allows for operating the spectrometer in a mode where different SBCAs, tailored to different elements and/or emission lines, are installed in different stacks. For these experiments, each SBCA group is aligned separately, and the corresponding configuration is then stored in the spectrometer configuration manager. The user can then easily manually switch between the configurations to operate the spectrometer in the chosen spectrometer mode, one at a time. The switching involves the formal selection of the configuration and an adjustment to the mechanical position of the spectrometer parts. Furthermore, the GUI has an option to set up spectrometer scans using the stored configurations that will be switched automatically when needed. This allows setting up complex multi-element and/or emission line scan sequences that can be run without user intervention.

## 5. Spectrometer data acquisition

Once aligned, the spectrometer can be used for several kinds of measurements. For HERFD-XAS measurements, the spectrometer is set to the energy of interest and the scan is performed as a function of the monochromator energy. Similarly, for RIXS, a set of HERFD-XAS scans are recorded, each with the spectrometer aligned to a preset energy. The HERFD-XAS scanning is performed continuously: the monochromator energy is moved according to the predefined trajectory and the data, including monochromator encoder position, incident beam intensity, transmission, fluorescence and HERFD-XAS signals, are recorded asynchronously in parallel. To measure the HERFD-XAS signal, the detector, either Pilatus 100K or SDD, is triggered using an external pulse generator. The HERFD-XAS signal is then computed as a sum of counts within the relevant ROI. The data streams are then cross-correlated in time based on the time stamps provided by the globally synchronized event receiver and obtained signals are resampled onto the user-defined energy grid. A detailed description of the ISS data acquisition and signal processing is described in the literature (Leshchev *et al.*, 2022 ▸). For HERFD-XAS scanning, the user can precisely define the total duration of the scan, time distribution between different parts of the spectrum, as well as the direction of the scan, *i.e.* energy-up or energy-down. These parameters are tailored to the sample based on the susceptibility to radiation-induced changes and the position and relevance of spectroscopic features. Typical HERFD-XAS scans are performed on time scales between a few seconds and a minute. The trigger rate of the detector is chosen so that the signal is oversampled in the relevant spectroscopic regions, thus keeping the spectral resolution optimal. On the other hand, setting the trigger rate to a high value decreases the efficiency of the measurement due to the readout time, typically ≤1 ms. In practice, we find that frame rates of 25–100 Hz work well for most applications.

**Figure 5:**
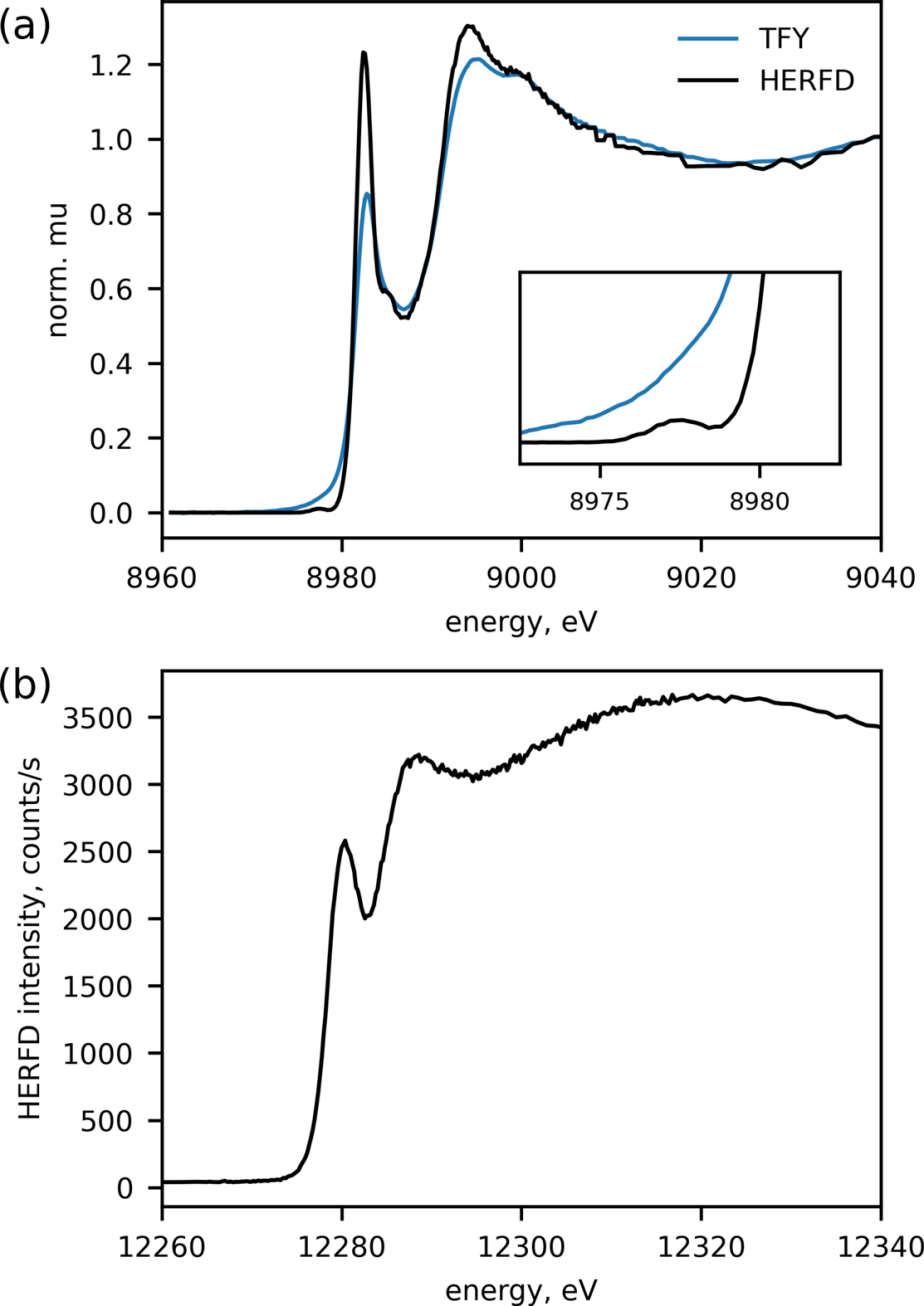
Examples of HERFD-XANES spectra recorded at ISS. (*a*) HERFD and TFY data collected on Cu-zeolite samples with ∼1.5 wt% metal loading; the single scan was measured in 5.7 s. (*b*) An example of a HERFD-XANES spectrum at the Hg *L*_3_ edge on a sample of muscle tissue from an Atlantic bottle nose dolphin (*T. truncates*) containing an Hg concentration of 19.3 p.p.m. by weight. The measurement details are given in the main text.

Fig. 5 ▸ shows examples of measurements performed on realistic systems. Fig. 5 ▸(*a*) shows a comparison of HERFD and total fluorescence yield (TFY) data on a Cu-zeolite sample with ∼1.5 wt% of Cu loading measured at 200°C under a mixture of NH_3_ and NO gases. The spectrometer was equipped with five Si(444) analyzers with a radius of curvature of 1000 mm tuned to the maximum of the Cu *K*α_1_ line. The data shown were recorded in a single scan with a duration of 5.7 s. The intensity of pre-edge features as observed in the HERFD spectra can be used to isolate Cu(I)/Cu(II) redox kinetics in this system. Fig. 5 ▸(*b*) shows an example of a HERFD-XANES spectrum at the Hg *L*_3_ edge on a sample of muscle tissue from an Atlantic bottle nose dolphin (*Tursiops truncates*) with a total Hg concentration of 19.3 p.p.m. by weight. The tissue was freeze dried, homogenized, and pressed as a pellet. During the measurement, the pellet was kept below 10 K in a closed-loop He cryostat. The spectrometer was equipped with an SDD detector and five Si(555) analyzers with a radius of curvature of 500 mm tuned to the maximum of the Hg *L*α line. The scan duration and the detector trigger rate were set to 8 s and 50 Hz, respectively. A new spot on the sample was used for each scan; a total of 236 scans were collected to compute the average signal.

For XES measurements, the monochromator energy is set to the energy of interest, typically above the absorption edge of the element of interest, and the scan is performed by varying the spectrometer energy in step scan mode. To avoid radiation-induced damage to the sample, a fast shutter blocks the incident beam during the spectrometer’s mechanical movements between the exposures.

XES scanning with Johann spectrometers tends to have low efficiency due to the mechanical motion overhead resulting in a significant dead time between the points, of the order of a second per energy step. Similar to XAS scanning, an increase in measurement efficiency could be achieved by performing scans continuously, which is a non-trivial task due to the large number of mechanical degrees of freedom that need to move synchronously. At ISS, when operating with all five analyzers, changing the spectrometer energy involves the adjustment of a number of motors, including the horizontal SBCA assembly stage, pitch of the central crystal, two rotations and two translation stages for the side crystals with *i* = (±1, ±2), and two rotations and a translation of the detector. Depending on the miscut and orientation of the central SBCA, its roll angle may also require an adjustment, bringing the total number of moving axes to 22. Modern motion-control solutions allow for highly coordinated multi-axis movements and have been implemented in synchrotron beamline settings, for example for XAS scanning with a monochromator and undulator gap moving in tandem (Hidas *et al.*, 2022 ▸). Despite this progress, implemented solutions typically involve coordinated motions of a few axes, whereas here one needs to orchestrate 22 axes distributed across several motion controllers from different vendors. While such an implementation is a part of the ISS long-term development plan, as an interim solution, we have recently implemented a continuous scanning scheme that takes advantage of the ISS asynchronous data-acquisition approach, as well as the usage of an area detector as an integral part of the spectrometer.

**Figure 6:**
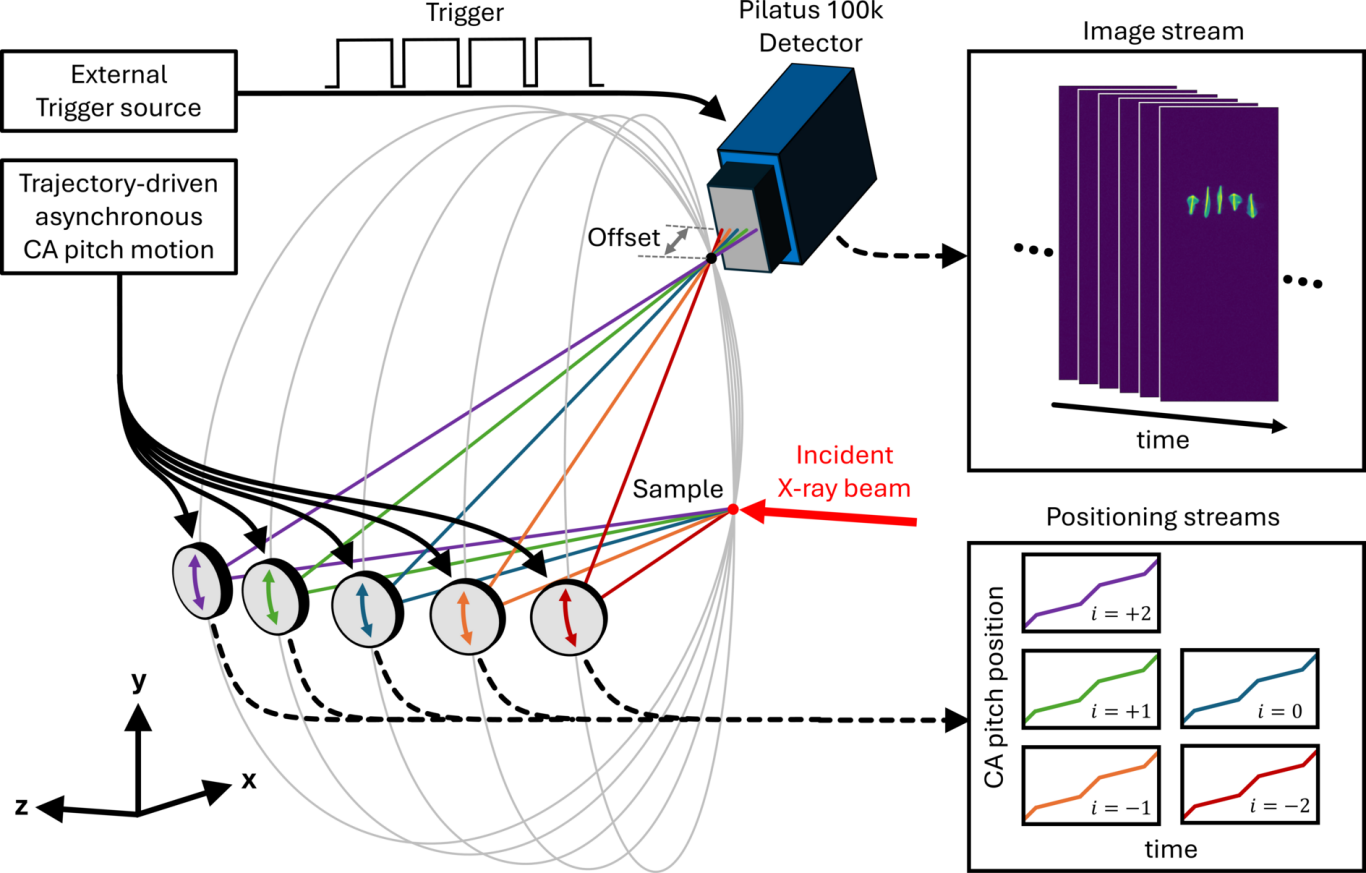
Implementation of continuous XES scanning. At the beginning, the spectrometer is set to the relevant emission energy, typically in the middle of the scanning range. The detector is then positioned outside of the intersecting Rowland circles to separate the individual SBCA reflections, as explained in the main text. The order of SBCA reflections is inverted on the detector sensor with respect to the SBCA arrangement. The scan is performed by asynchronously moving the SBCA pitch motors according to the user-supplied trajectory while collecting images with the Pilatus 100K detector using an external trigger source. The data consist of the image stream, individual SBCA positioning streams and ion-chamber currents (not shown). The coordinate system of the beamline is given for reference.

The continuous XES scanning implementation scheme is shown in Fig. 6 ▸. Conceptually, the scanning is based on a continuous asynchronous movement of individual SBCA pitch stages, which serve as pr­oxies for SBCA Bragg angles, while the rest of the spectrometer components remain idle. The XES signal is collected with the Pilatus 100K detector, with its sensor oriented along the meridional plane of the central crystal, allowing for a broader Bragg angle coverage for a given detector position. To avoid the loss of resolution in the recorded signals due to mismatches between SBCA Bragg angles during the asynchronous motion, signals from individual SBCAs are measured independently. To separate the SBCA foci from each other on the sensor, the detector is moved outside of the Rowland circle by 50–100 mm, depending on the Bragg angle and SBCA quality, prior to the scan. In this configuration, the SBCA reflections have sagittal focusing and are displaced horizontally with respect to each other. During the pitch motion, each SBCA focus moves along their respective meridional planes. Thus, the central SBCA (*i* = 0) focus moves vertically on the detector sensor, whereas the other SBCA foci will move at an angle with respect to the vertical plane. Thus, additional adjustments of the individual SBCA roll angles may be required to ensure that all foci remain separated, as well as within the detector sensor, across the entire SBCA pitch scan range. In practice, the described adjustments to the positioning of the spectrometer components are performed once the spectrometer is set to a specific energy, typically in the middle of the relevant energy range. Following the setup, the XES scan is executed by starting the SBCA pitch movement according to the user-defined trajectory, represented as a set of energies and times spent between them. The trajectory-based approach allows one to optimize the distribution of the time between the peaks and tails in the XES spectrum, maximizing the signal-to-noise ratio for the most relevant spectral features. During the scan, the pitch positions, detector images and ion-chamber signals are recorded as a function of time to be used for the signal reconstruction after the scan.

**Figure 7:**
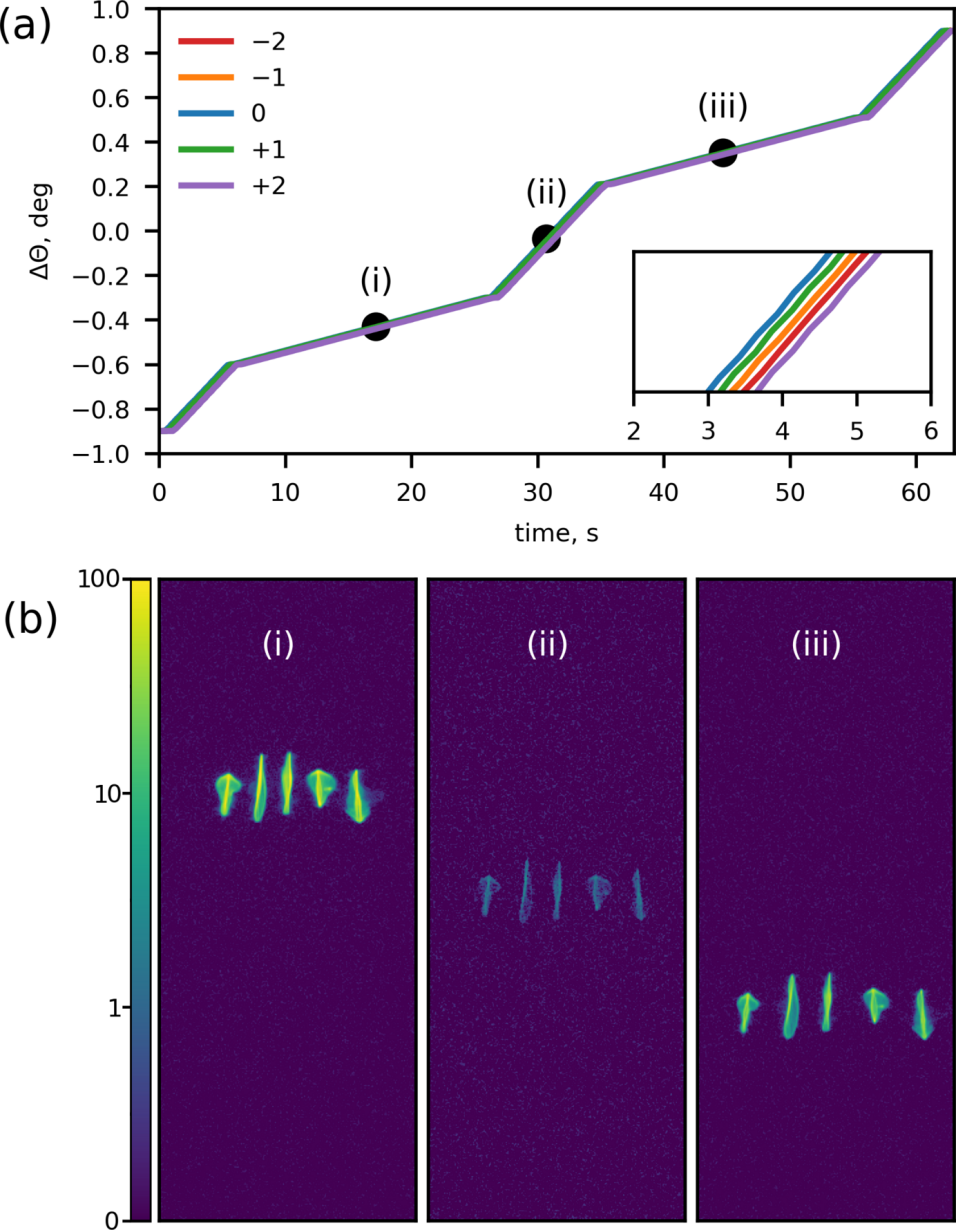
Raw data recorded during a continuous XES scan performed on the Cu *K*α emission lines. (*a*) The recorded SBCA pitch positions as a function of time. The positions are shown as deviations from the position at which the spectrometer was configured prior to the continuous scan (Bragg angle of 79.81° corresponding to 8035 eV). The SBCA pitch was configured to move with a slower speed in the regions corresponding to the *K*α_1_ and *K*α_2_ peaks and faster otherwise. The inset shows the recorded pitch positions during the first few seconds and shows the 150–200 ms delay between the individual SBCA pitch positions arising due to the asynchronous motion. Black circles display the representative times selected for the Pilatus 100K images shown in panel (*b*). Labels (i), (ii) and (iii) correspond to the peak of the *K*α_1_ line, a point in between the *K*α_1_ and *K*α_2_ lines, and the peak of the *K*α_2_ line, respectively. (*b*) Recorded Pilatus 100K images demonstrating the changes in the position and the intensity of the SBCA reflections during the SBCA pitch motion.

Fig. 7 ▸ shows representative data collected during a continuous XES scan. As an example, we show the *K*α emission data recorded on Cu foil using five Si(444) SBCAs. Prior to scanning, the spectrometer was set to a Bragg angle of 79.81° corresponding to 8035 eV, *i.e.* in between the *K*α_1_ and *K*α_2_ peaks, and the area detector was moved 75 mm out of the Rowland circle. The trajectory consisted of several regions, with slower SBCA pitch velocity across the emission peaks and faster velocity outside of them, with a total duration of 62 s. The recorded SBCA pitch positions are shown in Fig. 7 ▸(*a*). The inset demonstrates a 150–200 ms delay between individual SBCA pitch positions at any given time due to the asynchronous motion. Images recorded during the scan demonstrate how SBCA reflections change the intensity and move across the detector as SBCA pitch positions change [Fig. 7 ▸(*b*)].

**Figure 8:**
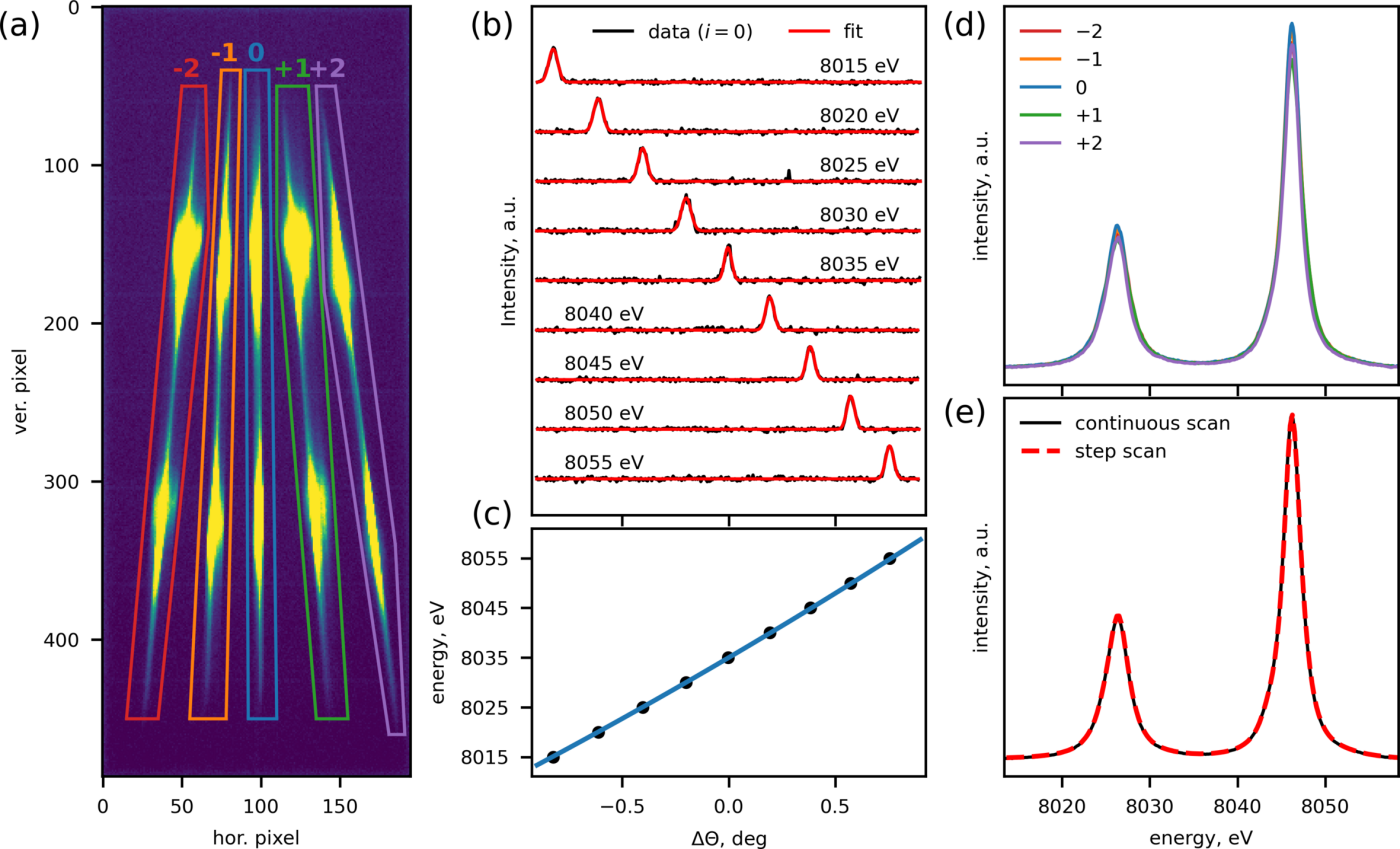
Processing of the continuous XES scan data. (*a*) A sum of all images recorded during the scan, demonstrating the footprint of each SBCA reflection as they traverse the detector. The footprints help to define polygonal ROIs that are then used to compute individual SBCA XES intensities as a function of time using recorded images. The polygonal ROIs are marked with their corresponding SBCA index. (*b*) A set of elastic scans performed to experimentally determine the conversion between the pitch angle and energy for the central SBCA (*i* = 0). The pitch data are shown as deviations from the central positions that correspond to the Bragg angle of 79.81° (8035 eV). Prior to scanning, the spectrometer was set to the Bragg angle of 79.81° corresponding to 8035 eV, *i.e.* in between the *K*α_1_ and *K*α_2_ peaks, and the area detector was moved 75 mm out of the Rowland circle. The black lines show elastic signal computed as the sum of intensities recorded by the pixels located within the corresponding polygonal ROI shown in (*a*) and normalized by the incoming beam intensity. By fitting the data with Gaussian peaks, shown as red lines, we determined the peak positions for the calibration. (*c*) Comparison of the experimentally determined conversion from SBCA pitch (*i* = 0) to energy (black circles) with a line computed based on the Bragg law. (*d*) XES signals obtained for individual SBCAs. (*e*) Comparison of the continuous XES scan with the step scan performed on the same Cu foil.

Fig. 8 ▸ shows the data-processing workflow for a continuous XES scan as well as a comparison with a traditional step scan. First, following the scan, the recorded images are processed to calculate signals coming from each SBCA. Since the foci of non-central SBCAs with *i* = (±1, ±2) move at an angle across the detector sensor during the scan, the simplest way to calculate the signal is to define a polygonal ROI for each SBCA focus and sum the intensities for the relevant pixels in each individual image. The ROIs are drawn around the footprint of each SBCA, which can be visualized by inspecting an image that combines all images taken during the scan, as shown in Fig. 8 ▸(*a*). In the case of a strong background, a better signal-to-noise ratio for XES signals could be achieved by using smaller ROIs positioned in sync with SBCA reflections in individual images; this procedure will be implemented in the future. Second, the data streams are interpolated onto the common time grid, similar to XAS scans. Third, individual SBCA pitch positions are converted to their corresponding emission energies. As a first approximation, the conversion of SBCA pitch positions to the emission energy can be done simply via the Bragg equation since the central emission energy and SBCA pitch positions are known. To verify that the simple conversion is valid, a set of 5–10 elastic scans are performed to calculate the calibration curve. To test the consistency between the approaches, we performed a set of elastic scans, using water as a sample, and fitted them with Gaussian peaks [Fig. 8 ▸(*b*)] to obtain the experimental calibration. While we find a good agreement between the Bragg equation and calibration based on elastic peaks [Fig. 8 ▸(*c*)], the simplified SBCA motion and additional adjustments to the spectrometer components may introduce offsets that are not accounted for in the simple Bragg equation. The potential offsets can be further amplified when operating at lower Bragg angles. Thus, in practice, it is better to employ experimental calibration based on elastic scattering to avoid any ambiguity in conversion of pitch positions to emission energy. Finally, the signals from individual SBCAs are binned onto a common emission energy grid with a step size of 0.2–0.3 eV and summed to yield the total measured XES intensity as a function of energy, as shown in Fig. 8 ▸(*d*). A good agreement between individual SBCA XES signal line shapes assures that the energy calibration is consistent across SBCAs. Minor discrepancies between the intensity of individual SBCA signals are observed, most likely due to the differences in the SBCA quality, as well as geometric factors, such as emission exit angles from the sample. Comparison of the data obtained via the continuous scanning method with the standard step scan shows a good agreement, indicating equivalence of the data measured using the two methods [Fig. 8 ▸(*e*)]. This also shows that the loss in the resolution due to the simplified single-axis motion for each individual SBCA is negligible. To further validate the continuous scanning procedure at lower Bragg angles, we performed a similar test by measuring Ni *K*β_1,3_ main emission lines and observed a good agreement between continuous and step scans (Section S7).

The results show that the continuous scanning approach using asynchronous SBCA motion presented here shows a route towards improving the efficiency of the XES measurements. However, although promising, the current approach has several drawbacks, which may limit its applicability for certain challenging systems. For example, in the case of samples with low concentrations of the element of interest embedded in high-*Z* matrices, the XES signal might be overwhelmed by elastic and/or fluorescence background, requiring the usage of the energy-discriminating SDD and thus step scanning. In addition, the current implementation is based on the high-level network-based control of the SBCA motion, which limits the scanning reproducibility at higher scanning speeds. Thus, presently, the highest pitch velocity can reach only 30 mdeg s^−1^, corresponding to ∼1 eV s^−1^ for Cu *K*α emission, thus limiting the applicability of the method to time-resolved studies. These limitations can be overcome by implementing the low-level control of the piezoelectric angular motions, which will allow increase of the scanning speed due to more reliable motion control, as well as enabling synchronous motion of the pitch and roll angles of all SBCAs, thus removing the need to separate the SBCA foci on the detector. While the former will improve the time resolution of the experiment, the latter will improve the signal-to-background ratio since all SBCA signals can be combined within the same ROI. Overall, this will be a stepping stone on the path to fully coordinated motion. Notably, the choice between continuous-scan and step-scan methods should be guided by the experiment’s specific needs. Continuous scans are ideal for radiation-sensitive samples and *operando* experiments where time resolution is crucial, as they allow faster data acquisition and minimize exposure. They also reduce dead time associated with motor motion, making better use of available photons. However, as mentioned above, step scans may still be suitable for experiments requiring usage of the SDD detector due to its ability to better separate signal from the background, provided that the dead time does not significantly impact the measurement.

## 6. Conclusions

This article presents a five-analyzer Johann spectrometer recently commissioned at the ISS beamline at NSLS-II. It details the instrument’s mechanical design, motion control, alignment procedures and data-acquisition scheme. The alignment protocol allows performance evaluation of the spectrometer using either elastic scattering or emission signals, each of which has its own pros and cons. For HERFD-XAS measurements, the article emphasizes the importance of rapid continuous scanning and fine trajectory control for radiation-sensitive samples. For XES measurements, it demonstrates enhanced data-collection efficiency through a continuous data-acquisition scheme based on asynchronous SBCA motion. As the first step towards fully synchronized spectrometer motion, the current implementation paves the way for combining the high signal-to-background ratio of Johann geometry with efficient data acquisition, which will enable exploration of even more dilute and/or radiation-sensitive systems, as well as time-resolved experiments. Finally, the article discusses how access to both area and energy-dispersive detectors allows tailoring of the instrument performance to specific experimental needs.

## 7. Related literature

The following reference, not cited in the main body of the paper, has been cited in the supporting information: Horn & Johnson (1985 ▸).

## Supplementary Material

Supporting information. DOI: 10.1107/S1600577524009342/ok5123sup1.pdf
