# Supplementary material for: Five-analyzer Johann spectrometer for hard X-ray photon-in/photon-out spectroscopy at the Inner Shell Spectroscopy beamline at NSLS-II: design, alignment and data acquisition
Source: J Synchrotron Radiat. 2024 Oct 30;31(Pt 6):1609–21. doi: 10.1107/S1600577524009342 (PMC11542649; doi:10.1107/S1600577524009342)
Supplement: Supplementary file 1 [file s-31-01609-sup1.pdf]

JOURNAL OF SYNCHROTRON RADIATION

Volume 32 (2025)

Supporting information for article:

Five-analyzer Johann spectrometer for hard X-ray photon-in/photon-out spectroscopy at the Inner Shell Spectroscopy beamline at NSLS-II: design, alignment and data acquisition

Akhil Tayal, David Scott Coburn, Donald Abel, Max Rakitin, Oksana Ivashkevych, Jakub Wlodek, Dominik Wierzbicki, Weihe Xu, Evgeny Nazaretski, Eli Stavitski and Denis Leshchev

S1. Detector Motion

The figure shows the equation of motions to realize detector motion using two goniometer and one translation stage.

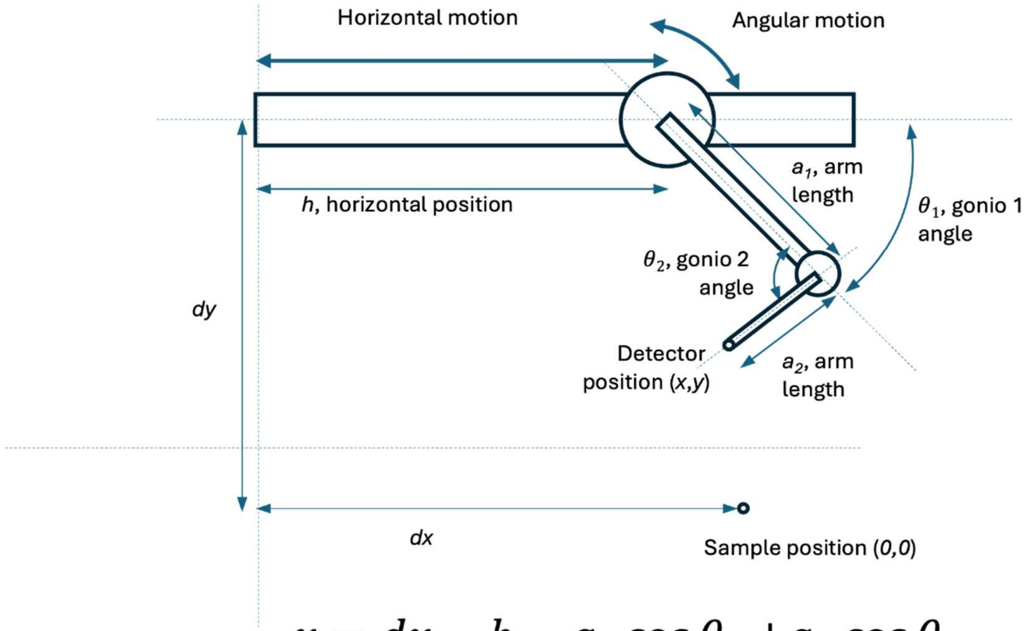

$$x = dx - h - a_1 \cos\theta_1 + a_2 \cos\theta_2$$

$$y = dy - a_1 \sin\theta_1 - a_2 \sin\theta_2$$

Figure S1 Schematic of detector motion realized with two goniometer stage and one translation state.

S2. Currently available analyzers for Johann spectrometer

Figure S2 shows available analyzers and their respective energy ranges for 1m SBCAs. For 0.5m configuration, we currently have Si-111 and Si-311 SBCAs. In the figure the horizontal scale represents the energy range covered by all the SBACs currently available at the beamline. The vertical lines indicate the energies of various emission lines, as indexed. The horizontal blue and red bars illustrate the Bragg angle range for the corresponding SBACs, along with the energy range they cover, as labeled. The length of blue and red bars represent the Bragg angle range from 88-65˚.

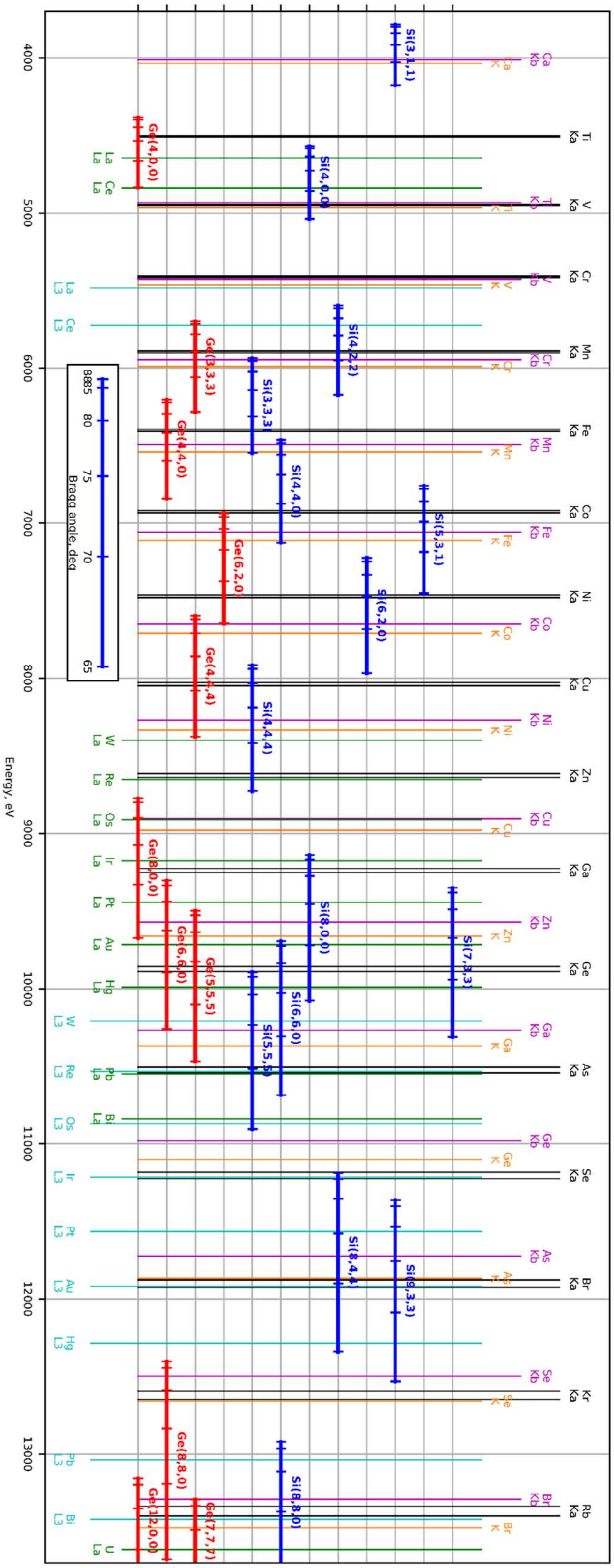

Figure S2 Atlas of available analyzers and their respective energy ranges for 1m SBCAs.

S3. Angular positioning repeatability of piezoelectric motor stages

The angular positioning repeatability of the piezoelectric stages for the analyzer pitch motion was measured by installing a laser interferometer (FPS3010, Attocube Inc.) with two collimating sensor heads, separated by 15 mm, next to a crystal analyzer placed in the spectrometer socket. The pitch changes as a function of time were estimated based on the difference in the distances measured by the sensor heads. To measure the deviation from the target position, the stage was placed onto a target position, which was followed by a sequence of positive (forward) and negative (backward) displacements where the stage was deviated from and then returned to the target position. The sequence was repeated 50-100 times to collect statistics. During the motion, the angle was continuously measured using the interferometer and recorded as a function of time with a sampling rate of 381.5 Hz. The data was analyzed to locate stationary ranges following the return of the stage to the target position. The deviation from the target position was calculated as an average of 50 points around the middle of each stationary range. The measurements were repeated for different motion amplitudes, including 10, 30, and 100 millidegrees. Figure S3 shows a few cycles of a typical angular motion used here, as well as the spread of the recorded deviations from the target angle. Overall, we find that the bi-directional repeatability of the motion lies within ±0.2 millidegrees from the target position.

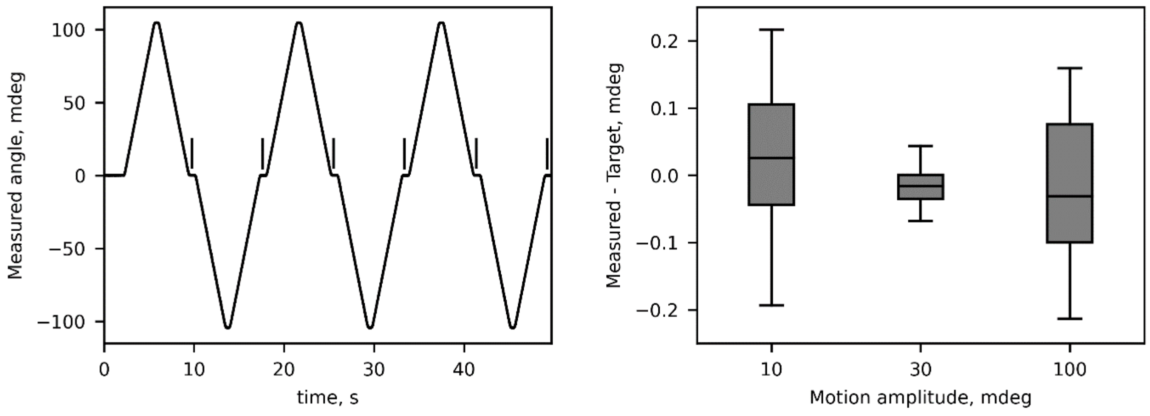

Figure S3 (left) A typical motion profile used to assess the repeatability of the piezoelectric stage for the analyzer pitch motion. The vertical lines schematically show the stationary regions used to estimate the deviation from the target. (right) box plot of the recorded deviations from the target position as a function of the motion amplitude.

S4. Determination of the optimal Rowland circle radius using different methods

To evaluate the reproducibility between different methods of the spectrometer alignment, elastic scattering and emission signals were measured as a function of the Rowland circle radius R. As explained in the main text, the spectrometer was configured to operate with five Si(444) analyzers placed at a Bragg angle of 79.43° corresponding to 8046 eV. For elastic scattering, we used a 1 mm Kapton tube with deionized water and Cu foil. For emission, we used Kα_1 peak measured on Cu foil. The signal intensity was calculated using the total counts, measured within the relevant region of interest of the Pilatus 100k detector, normalized by the intensity of the incoming beam measured using an ion chamber. The data was analyzed to estimate the peak maximum intensity and the full width at half maximum (FWHM). The results for all methods, metrics, and samples are shown in Figure S4. The optimal values of the Rowland circle radius obtained from different methods are shown in Table S1. The uncertainty in the optimal R values were estimated by calculating the distance at which the changes of the measured metric (FWHM, max. intensity) reach 10% from the optimal value. We observe that regardless of the measured quantity (elastic scattering, emission) and the nature of the sample, the derived optimal R-values, obtained based on the peak maximum intensity, agree with each other. When FWHM is used to calculate the optimal R value, we observe a similar agreement with the exception of crystal analyzers with $i = +1$ and $i = -2$, which show disagreement between elastic and emission approaches on the order of 4-6 mm and 6-7 mm, respectively. A possible reason for this discrepancy is the presence of the low-energy tail observed in the elastic scattering scans performed using these crystal analyzers (Figure 3b of the main text) thus potentially biasing FWHM as a metric.

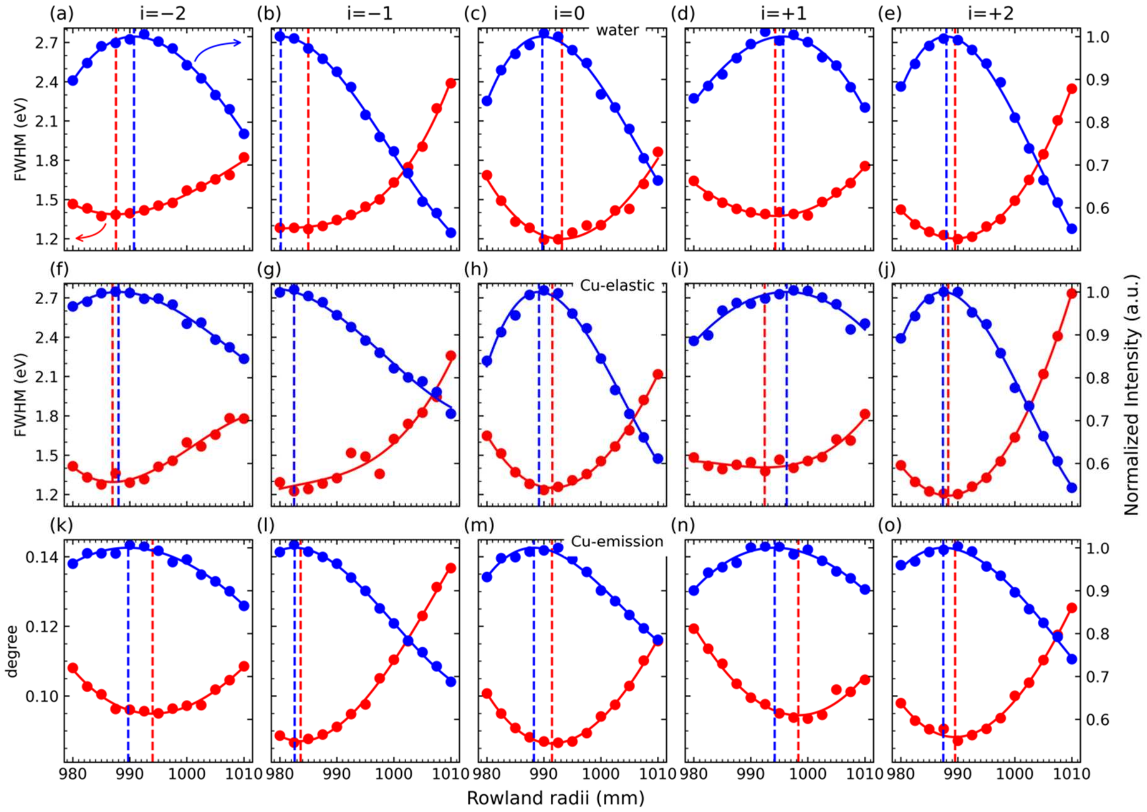

Figure S4 Elastic (a-j) and emission (k-o) alignment scans with water (a-e) and Cu-foil (f-o) for different crystal analyzers as indicated. Blue and red cirlces show variations in peak maximum intensity and FWHM, respectively, as a function of Rowland circle radius R. The solid lines show third-order polynomial fits to the raw data and the dashed lines show the extrema of the fitted curves. The extremum for each curve was found by computing the roots of the derivative of the fitted polynomial (Horn & Johnson, 1985). If the extremum fell outside of the measured range of Rowland cirlce radii, then we used the extremum position determined based on the raw data.

Table S1 Optimal Rowland circle radius, R-value (mm), for different crystal analyzers based on the peak maximum intensity and FWHM determined from elastic data measured on water and Cu foil and emission data measured on Cu foil.

Crystal analyzer index	Peak FWHM			Peak maximum intensity		
	Water	Cu-elastic	Cu-emission	Water	Cu-elastic	Cu-emission
i=−2	987.6±2	986.9±2	994.0±2	990.7±2	988.0±2	989.7±2
i=−1	985.0±2	982.5±2	983.6±2	980.2±2	982.5±2	982.6±2
i= 0	993.2±2	991.5±2	991.4±2	989.7±2	989.1±2	988.2±2
i=+1	994.3±2	992.4±2	998.3±2	995.7±2	996.3±2	994.2±2
i=+2	989.5±2	988.3±2	989.5±2	988±2	987.4±2	987.4±2

S5. Elastic scattering signal measured off different objects

To understand the effect of different types of scatterers on the spectrometer resolution and energy calibration, the elastic alignment scans were performed using five types of scatterers namely: CeO_2, Cu-foil, LaB_6, water, and zeolite. Figure S5 shows the elastic data recorded using different scatterers, as well as the peak centroid and FWHM values as a function of the scatterer type. The data shows that for a given crystal analyzer peak FWHM can deviate by ±0.1 eV (Fig. S5(f)), and the peak centroid position (Fig. S5(g)) by ~ ±0.05eV depending on the scatter type.

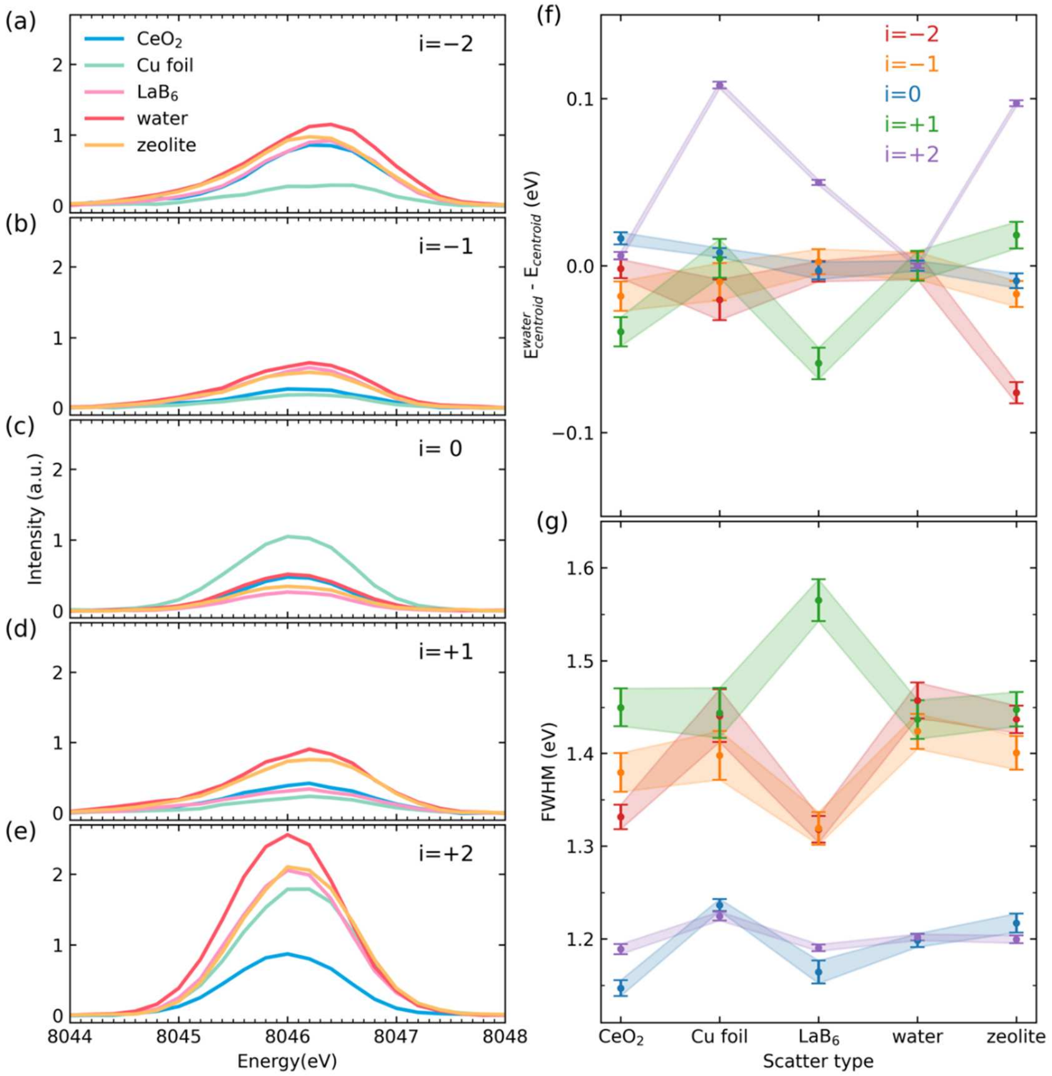

Figure S5 (a-e) Elastic scan performed at 8046 eV line with different scatterer as indicated for different crystal analyzers. (f) Peak centroid relative to water for different scatterers and crystal analyzers. (g) FWHM values for different scatter and crystal analyzers.

S6. Assessing the crystal analyzer illumination with elastically scattered X-rays

To see visualize analyzer surface illumination by different types of scatterers, the Pilatus 100k detector was placed in front of crystal analyzers with $i = 0, +1, +2$ (Fig. S6). The active area of the detector (83.8 x 33.5 mm^2) was oriented horizontally and placed at about ~300 mm in front of the crystals. The diffraction patterns were recorded with the incident beam focused on the sample position to 100 μm (FWHM) spot using a polycapillary optic and the monochromator energy set to 8046 eV. The crystalline samples such as CeO_2, Cu foil, and LaB_6 produce strong diffraction patterns which result in a non-uniform illumination of the analyzer surfaces (Figure S7(d-l)). On the other hand, water and zeolite (Figure S7(m-r)) produce no intense diffraction peaks and thus result in a more unform illumination of the analyzer surfaces. We note that zeolite is an ordered material that tends to produce multiple peaks with lower intensity when compared to other materials, e.g. CeO_2 (Figure S7(d-f) and S7(p-r)) which produce a smaller number of strong peaks. This is why the scattering image measured on zeolite does not show characteristic peaks. The results demonstrate that the diffraction patterns produced by the sample affect the uniformity of the crystal analyzer surface illumination, which results in the variations of the peak position and FWHM discussed in the note S3 and the main text. Furthermore, we observe that the specifics of the diffraction patterns affect the peak intensity observed in the elastic scans (Figure S7(a-c)). For example, in case of Cu foil, there are strong diffraction rings cast towards analyzers with i=0 and i=+2 (Figure S7(g, i)), and no diffraction observed for analyzer with i=+1(Figure S7(h)). The presence of the diffraction rings correlates with the higher elastic peak intensity observed for analyzers with i=0 and i=+2 (Figure S7(a, c)) when compared to i=+1 (Figure S7(b)). Finally, the same setup was used to measure diffraction using an unfocused and better-collimated beam without the polycapillary optic (Figure S8). The collimated beam tends to produce sharper diffraction patterns further exacerbating the inhomogeneity of the crystal analyzer illumination.

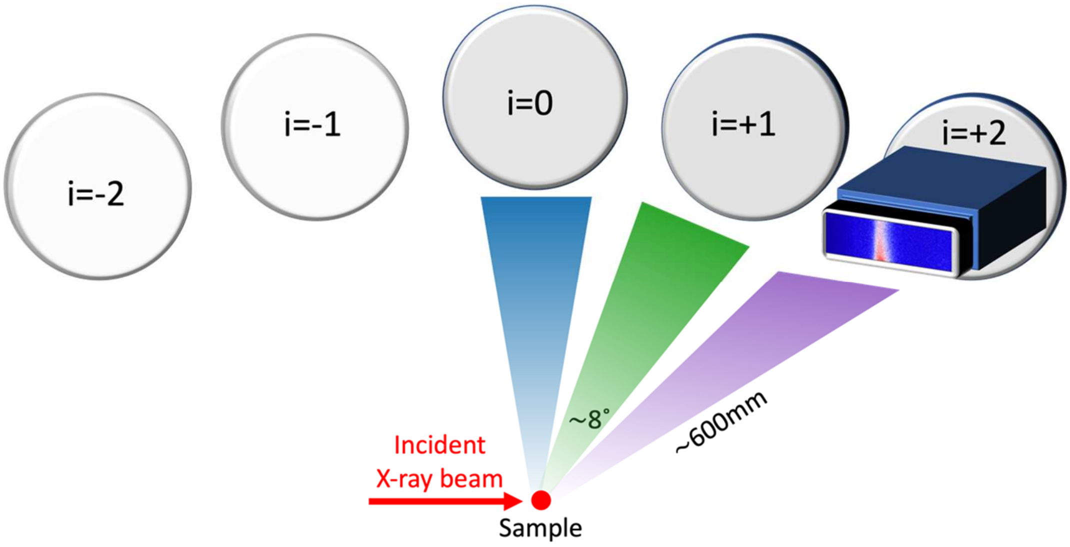

Figure S6 Schematic of the setup to measure scattering from the sample at various crystal analyzer positions.

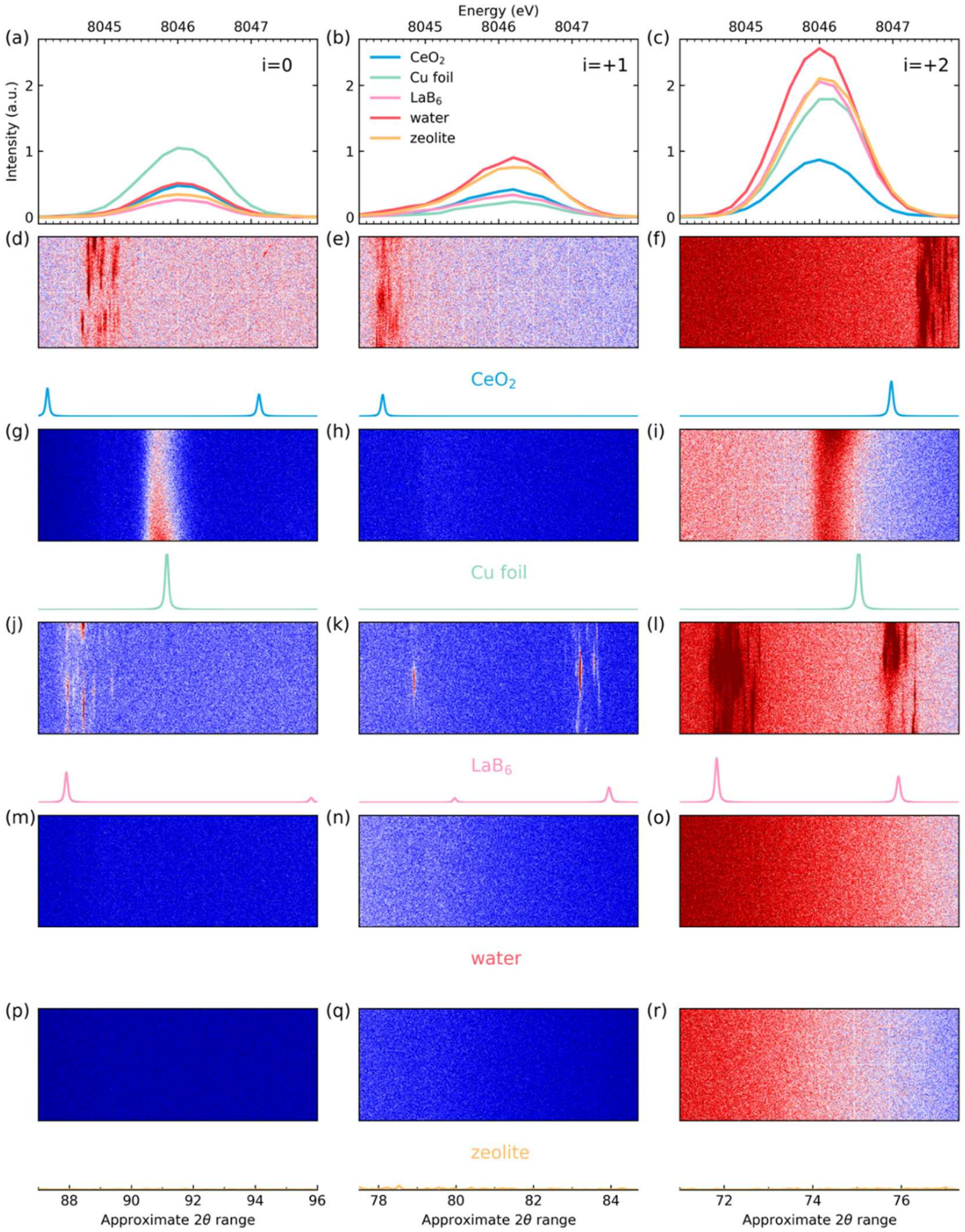

Figure S7 Intensity of elastic peak (a-c) and scatter image seen by a Pilatus 100k detector placed in front of three crystal analyzers i=0, +1, +2 with different scatter as indicated. For comparison a simulated diffraction pattern expected from different scatter is also provided. The detector is roughly positioned in front of the crystal, covering an approximate 2θ range as indicated.

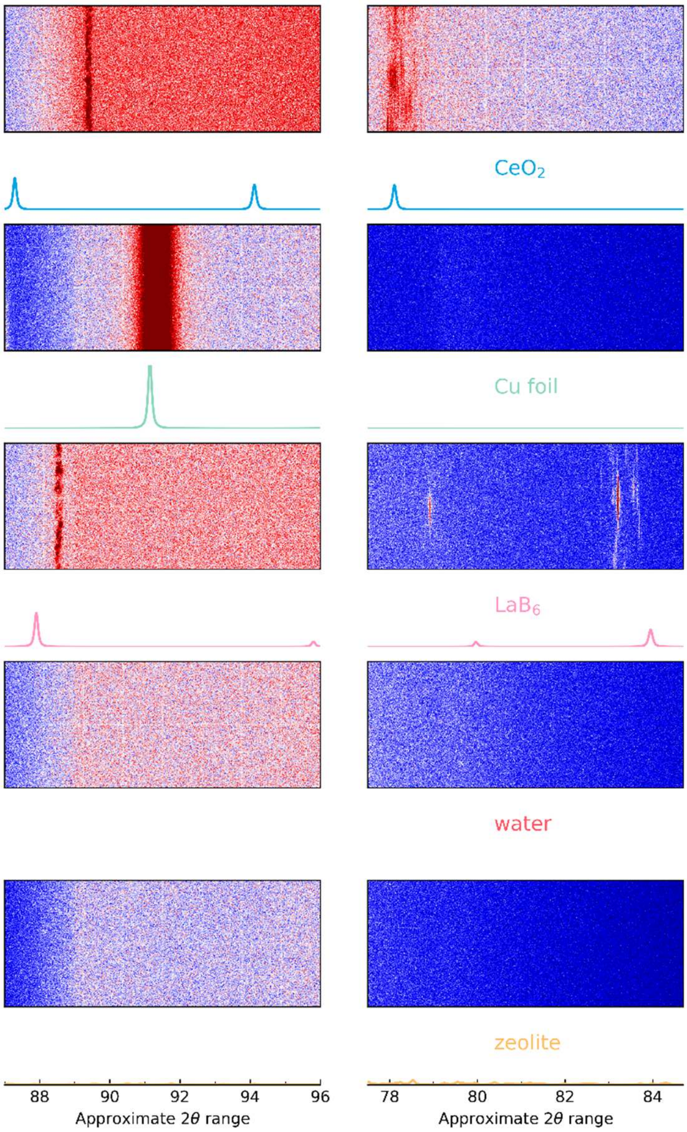

Figure S8 Diffraction patterns measured by a 2D detector placed in front of two crystal analyzers with i=0, +1 with different scatterers as indicated with the unfocused and collimated beam. For comparison a simulated diffraction pattern is also provided.

S7. Continuous X-ray emission scan of Ni Kβ line

To explore the performance of the continuous scanning at lower Bragg angles, we tested the approach by measuring $K\beta_{1,3}$ main line on Ni foil using 5 Si(444) analyzers configured at Bragg angle of 73.17 degrees, corresponding to the energy of 8262 eV in the vicinity of the main peak. The detector was moved out of the Rowland circle by ~75 mm. The scanning was done using a linear trajectory spanning ±0.45 degrees for the pitch motion. By measuring additional set of elastic peaks, the pitch positions were

converted to emission energy. The results of the measurement are shown in Figure S9. Comparison of the sum of the signals from the individual analyzers shows a good agreement with the step scan, indicating validity of the method.

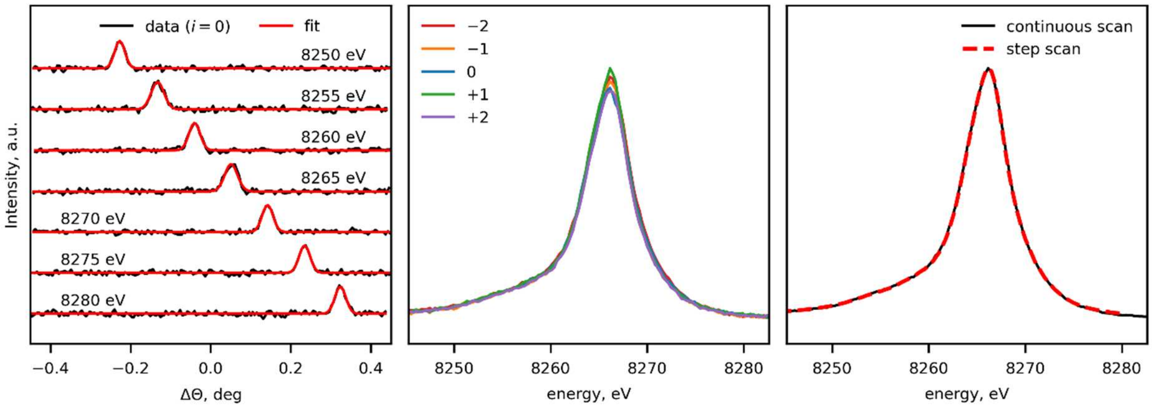

Figure S9 Continuous scanning of Ni Kb1,3 main line on Ni foil. (left) series of elastic scans and their corresponding fitting for the central crystal with $i = 0$. (middle) Comparison of the XES spectra measured by different analyzers. (right) Comparison of the emission spectra measured using continuous scan and step scan.

S8. References

Horn, R. A. & Johnson, C. R. (1985). *Matrix Analysis*. Cambridge: Cambridge University Press.
